# Differential Effects of a Glucagon-Like Peptide 1 Receptor Agonist in Non-Alcoholic Fatty Liver Disease and in Response to Hepatectomy

**DOI:** 10.1038/s41598-018-33949-z

**Published:** 2018-11-07

**Authors:** M. Pilar Valdecantos, Laura Ruiz, Virginia Pardo, Luis Castro-Sanchez, Carmelo García-Monzón, Borja Lanzón, Javier Rupérez, Coral Barbas, Jaqueline Naylor, James L. Trevaskis, Joseph Grimsby, Cristina M. Rondinone, Ángela M. Valverde

**Affiliations:** 1 Instituto de Investigaciones Biomédicas Alberto Sols (Centro Mixto CSIC-UAM), Arturo Duperier 4, 28029 Madrid, Spain; 2 Centro de Investigación Biomédica en Red de Diabetes y Enfermedades Metabólicas Asociadas (CIBERdem), ISCIII, 28029 Madrid, Spain; 3 CONACyT-University of Colima, Colima, 28045 Mexico; 4 Liver Research Unit, Santa Cristina University Hospital, Instituto de Investigación Sanitaria Princesa, Madrid, 28009 Spain; 5 Centro de Investigación Biomédica en Red de Enfermedades Hepáticas y Digestivas (CIBERehd), ISCIII, 28029 Madrid, Spain; 6 Centre for Metabolomics and Bioanalysis (CEMBIO), Faculty of Pharmacy, Universidad San Pablo CEU, Campus Monteprincipe, Boadilla del Monte, 28668 Madrid, Spain; 7 MedImmune LTD, Cambridge, UK; 8 MedImmune LLC, Gaithersburg, MD 20878 USA

## Abstract

Non-alcoholic fatty liver disease (NAFLD) is associated with post-operative liver failure (PLF) and impaired liver regeneration. We investigated the effects of a glucagon-like peptide-1 (GLP-1) receptor agonist on NAFLD, PLF and liver regeneration in mice fed chow diet or methionine/choline-deficient diet (MCD) or high fat diet (HFD). Fc-GLP-1 decreased transaminases, reduced intrahepatic triglycerides (TG) and improved MCD-induced liver dysfuction. Macrophage/Kupffer cell-related markers were also reduced although Fc-GLP-1 increased expression of genes related to natural killer (NK), cytotoxic T lymphocytes and hepatic stellate cell (HSC) activation. After partial hepatectomy (PH), survival rates increased in mice receiving Fc-GLP-1 on chow or MCD diet. However, the benefit of Fc-GLP-1 on NASH-like features was attenuated 2 weeks post-PH and liver mass restoration was not improved. At this time-period, markers of NK cells and cytotoxic T lymphocytes were further elevated in Fc-GLP-1 treated mice. Increased HSC related gene expression in livers was observed together with decreased retinyl ester content and increased retinal and retinoic acid, reflecting HSC activation. Similar effects were found in mice fed HFD receiving Fc-GLP-1. Our results shed light on the differential effects of a long-acting GLP-1R agonist in improving NAFLD and PLF, but not enhancing liver regeneration in mice.

## Introduction

Non-alcoholic fatty liver disease (NAFLD), the most common cause of chronic liver disease in Western countries, has emerged as an important public health problem^1^. In its initial stages NAFLD is characterized by accumulation of ectopic fat in the liver. However, 15–20% of NAFLD patients progress to the more severe form of liver injury known as non-alcoholic steatohepatitis (NASH)^2^. NASH is characterized by recruitment of immune cells into the liver, mitochondrial dysfunction, endoplasmic reticulum (ER) and oxidative stress and defective autophagy, resulting in hepatocyte ballooning, apoptosis, and activation of hepatic stellate cells (HSC)^3,4^. These cells differentiate into myofibroblasts that produce collagen I, promoting liver fibrosis which affects approximately one-third of patients with NAFLD^5^. Although NAFLD is commonly diagnosed in obese or overweight individuals, prevalence of lean NAFLD has been described in different ethnic populations; these patients representing another significant end of the phenotypic spectrum of NAFLD^6^. Globally, rates of NAFLD in non-obese patients average between 10–30% in both Western and Eastern countries and are at markedly higher cardiovascular risk^7^. The lack of systematic screening for NAFLD has led to it being massively underdiagnosed^8^. Despite its impact on the public health system, there are no FDA approved drugs available for NAFLD/NASH. Thus, effective treatment options for NAFLD and associated hepatic, metabolic and cardiovascular disturbances are urgently needed^9^.

Gut hormone-based therapies, particularly those targeting glucagon like peptide-1 receptor (GLP-1R) are currently being used in both preclinical models and clinical trials of several stages of NAFLD linked to type 2 diabetes mellitus (T2DM)^10^. For instance, the GLP-1R agonist exendin-4 decreased hepatic steatosis and inflammation in mice with obesity or atherosclerosis^11,12^. Likewise, exendin-4 protected human hepatocytes against lipotoxic cell death by reducing ER stress and promoting autophagy^13^. Other studies reported that exendin-4 or AC3174, an exendin-4 analog, alleviated NASH in mice in a GLP-1R dependent manner^14,15^. More recently, the synergistic therapeutic effect of combined GLP-1R and farnesoid-X receptor agonism on features of NASH in mice was reported^16^. Regarding clinical studies, in a phase II 48-week randomized controlled trial, the GLP-1R agonist liraglutide induced resolution of NASH in both diabetic and nondiabetic patients, as well as improved steatosis and ballooning^17^. However, it was not possible to establish whether these histological effects were solely determined by reductions in body weight. Thus, it is premature to consider GLP-1R agonists to specifically treat liver disease in patients with NAFLD or NASH^10^.

Partial hepatectomy is an alternative to liver transplantation that circumvents organ shortage and long waiting periods associated with disease progression^18,19^. NASH-related cirrhosis is currently the third most common indication for liver transplantation in USA^20^. As liver regeneration is impaired in NASH patients^21^, new therapeutic approaches are needed to ensure a controlled and efficient regenerative response of the liver in this chronic disease. Considering that GLP-1R agonists are FDA approved drugs to treat T2DM and also ameliorate NASH, in the present study we evaluated the efficacy of a GLP-1R agonist in two mouse models: one model with NASH-like features (methionine and choline-deficient (MCD) diet), and a second high-fat model of metabolic syndrome and hepatic dysfunction. Furthermore, we studied for the first time the potential effects of Fc-GLP-1 on PLF and hepatic regeneration after PH during this pathological condition. The MCD model aimed to unravel the effects of the GLP-1R agonist on NASH-like features and restoration of liver mass after PH independently of its effects in reducing obesity.

## Results

### Treatment with Fc-GLP-1 attenuates the development of NAFLD in mice

**Figure 1:**
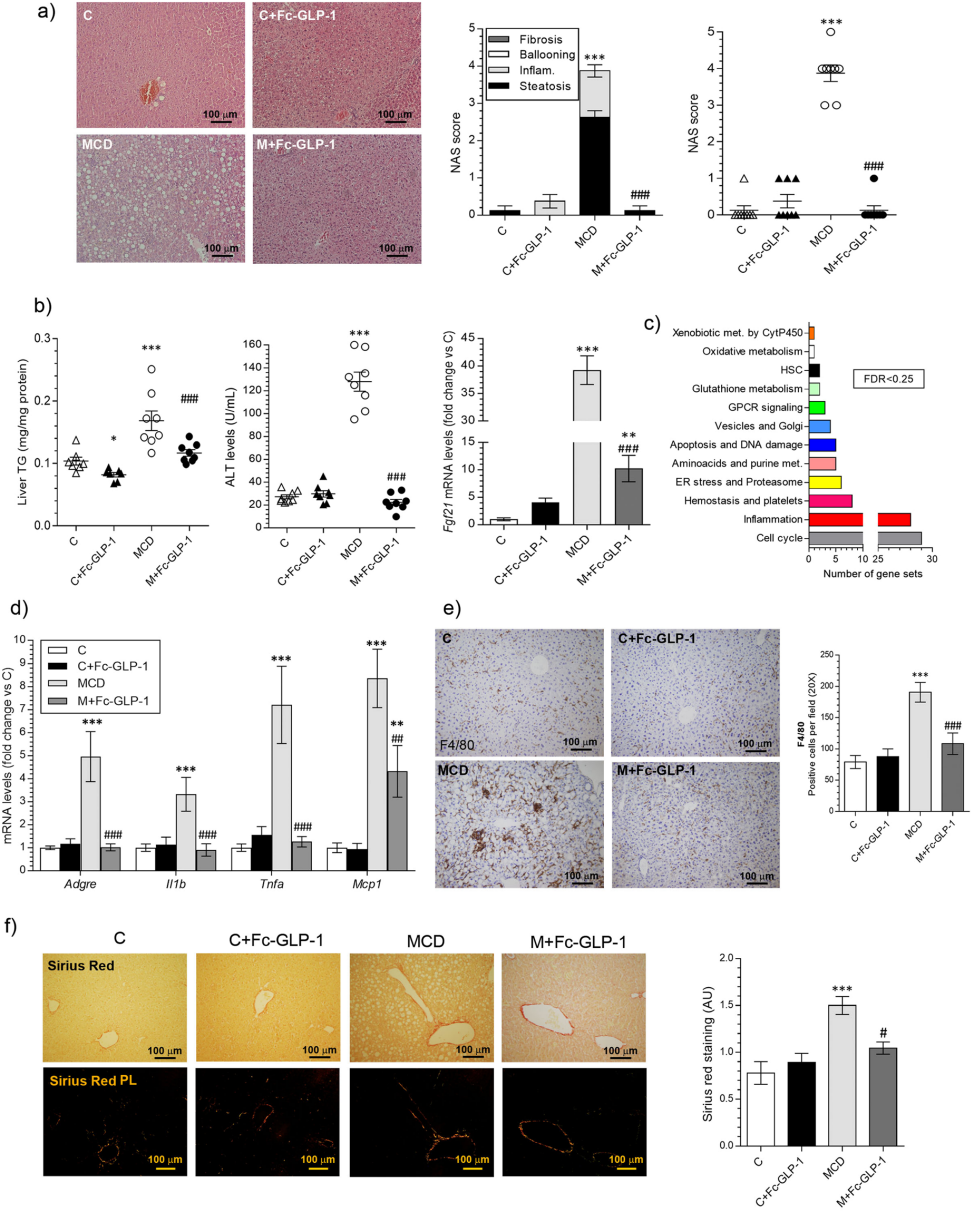
Treatment with a GLP-1R agonist alleviates NASH-like features in mice. (**a**) (Left panel) Representative H&E images from mice fed a standard chow diet (C) or MCD diet untreated or treated with Fc-GLP-1 (C+ Fc-GLP-1, M+Fc-GLP-1 respectively); (right panel) Histopathological evaluation of the NAS score (n = 8 mice/group). (**b**) Liver TG content, plasma ALT levels and hepatic *Fgf21* mRNA levels (**p = *0*.*0075*) (n = 8 mice/group). (**c**) Gene sets down-regulated in M + Fc- GLP-1 treated mice versus the MCD group (n = 4 mice/group). (**d**) Hepatic mRNA levels of proinflammatory cytokines in the different groups of mice (n = 6 mice/group). (**e**) Representative images of F4/80 immunohistochemistry in the different groups of mice and quantification of F4/80 positive cells (3 random fields per mice; n = 4 mice/group). (**f**) Representative images of Sirius Red staining in the different groups of mice and quantification of stained area (2 random fields per mice; n = 4 mice/group; ^#^*p* = *0*.*0482*). *p < *0*.*05*, **p < *0*.*01*, ***p < *0*.*001 vs C group*; ^##^*p* < *0*.*01*, ^###^*p* < *0*.*001 vs MCD group according to one-way ANOVA with post-hoc Bonferroni test*.

The effect of the GLP-1R agonist (hereafter referred as Fc-GLP-1) was first tested in mice fed a chow or MCD diet for 3 weeks (Supplementary Fig. 1A). Mice fed a MCD diet presented NASH-like features such as steatosis and inflammation^22,23^, assessed by the histopathological evaluation of the NAS score, intrahepatic TG accumulation, elevated ALT and increased *Fgf21* mRNA levels (Fig. 1a,b). Treatment with the GLP-1R agonist (M + Fc-GLP-1) prevented the accumulation of liver TG and normalized all of these parameters to values of the control group fed a chow diet (C).

Microarray gene expression analysis revealed that multiple gene sets were altered in the liver by the MCD diet and drug treatment (Supplementary Tables 1 and 2). In particular, cell cycle, inflammation, hemostasis and platelets, ER stress and proteasome, amino acid and purine metabolism, DNA damage and apoptosis gene sets were down-regulated in livers from M+Fc-GLP-1 versus MCD mice, suggesting that multiple pathways dysregulated in NASH were modulated by Fc-GLP-1 treatment (Fig. 1c and Supplementary Table 2). Among them, we validated the mRNA levels of the prinflammatory markers *Tnfa*, *Il1b and Adgre1* (encoding F4/80) (Fig. 1d), the later was also analyzed by immunohistochemistry (Fig. 1e). The elevations in these parameters found in the MCD group were reduced in M+Fc-GLP-1 animals to similar levels detected in mice fed a chow diet (C). Unexpectedly, *Mcp1* mRNA levels that were found highly elevated in the MCD group were only partly decreased by the Fc-GLP-1 treatment (Fig. 1d). We also performed Sirius Red staining and, as depicted in Fig. 1f, in agreement with the histopathological evaluation of the NAS score no evidences of fibrosis were found in mice fed a MCD diet for 3 weeks.

**Figure 2:**
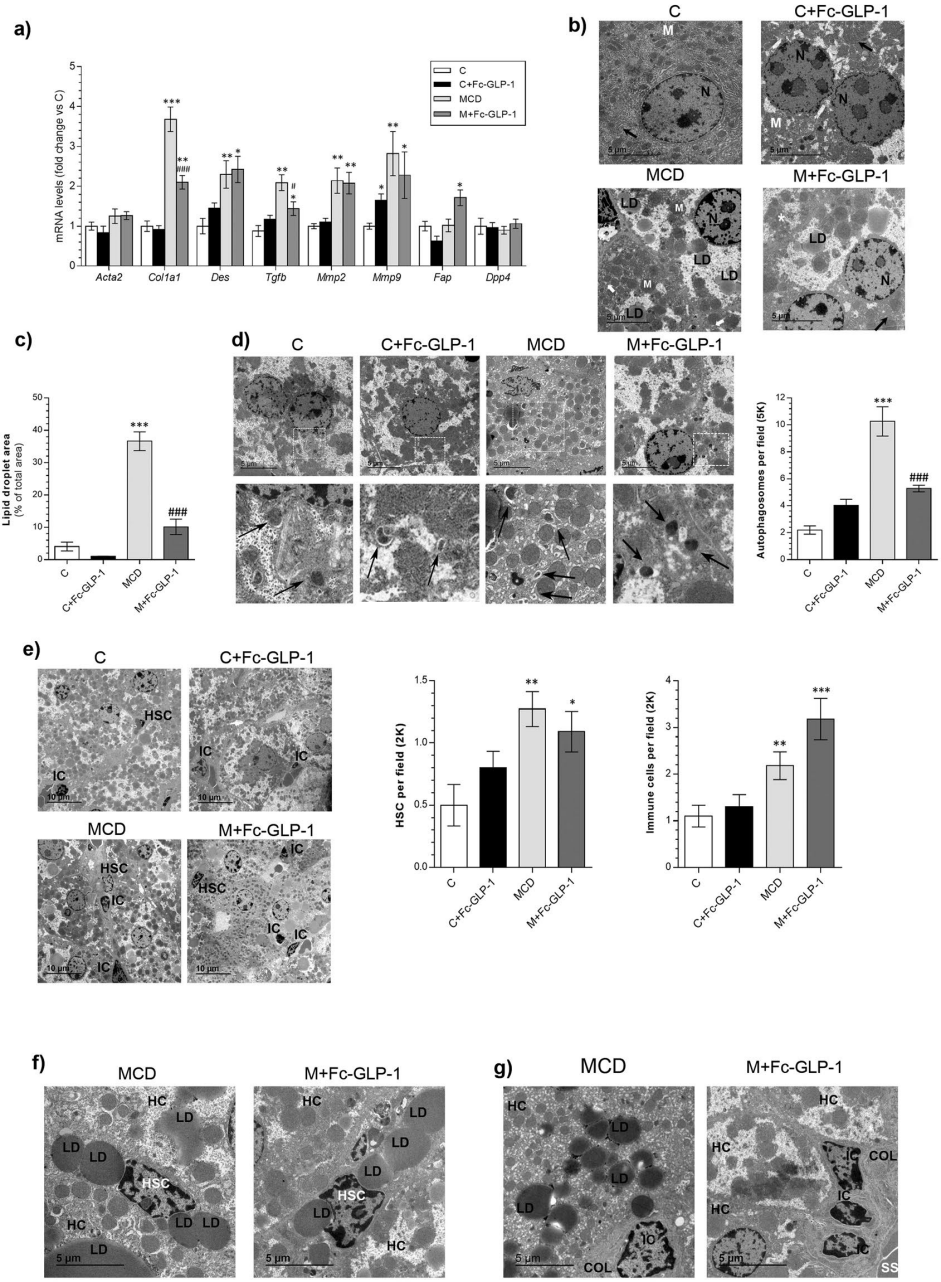
Effect of Fc-GLP-1 treatment in HSC activation in mice fed chow or MCD diet. (**a**) Hepatic mRNA levels of HSC activation-related genes in the different groups of mice (n = 6 mice/group). (**b**) Representative TEM images showing lipid droplets (LD), mitochondrion (M), nucleus (N), normal ER (black arrow) and dilated ER (white arrow) and (**c**) quantification of the area of lipid droplets in the TEM images (4 random fields per mice; n = 3 mice/group). (**d**) Representative TEM images and quantification of the autophagosomes (4 random fields per mice; n = 3 mice/group). (**e**) Representative TEM images and quantification of the immune cells (IC) (**p = *0*.*0052*) and hepatic stellate cells (HSC) (*p=0.022; **p = *0*.*0018*) in livers from the 4 groups of mice (5 random fields per mice; n = 3 mice/group). (**f**,**g**) Magnification of the images showing IC, HSC, hepatocytes (HC) and collagen fibers (COL) in livers from MCD and M+Fc-GLP-1 groups (n = 3 mice/group). SS (sinusoid). *p < *0*.*05*, **p < *0*.*01*, ***p < *0*.*001 vs C group*; ^#^*p* < *0*.*05*, ^###^*p* < *0*.*001 vs MCD group according to one-way ANOVA with post-hoc Bonfeni test*.

We also assessed the expression of genes present in HSC, which are activated in NASH^4^. Significant increases in *Col1a1*, *Des*, *Tgfb*, *Mmp2* and *Mmp9* mRNAs were detected in livers from MCD-fed mice compared to mice fed a chow diet (Fig. 2a). Treatment with Fc-GLP-1 only ameliorated the effect of MCD diet on *Col1a1* and *Tgfb* mRNAs. Moreover, mRNA encoding fibroblast activation protein (*Fap*), which has been found in activated HSC and myofibroblasts^4,24^, was elevated in the M+Fc-GLP-1 group. By contrast, *Dpp4* mRNA levels did not change among groups.

Transmission electronic microscopy (TEM) showed lipid droplets, rounded mitochondria and dilated ER in livers from the MCD group (Fig. 2b,c). The area of the lipid droplets, as well as the other NASH-like features, was reduced in the M+Fc-GLP-1 group. Likewise, the accummulation of autophagosomes detected in the MCD group was further reduced by the treatment with Fc-GLP-1 (Fig. 2d). Notably, some infiltration of non-parenchymal cells such as immune cells (IC) and HSC in the Disse space were visualized in livers from mice fed a MCD diet with or without Fc-GLP-1 treatment (Fig. 2e–g).

### Effects of Fc-GLP-1 in PLF and hepatic regeneration in mice fed chow or MCD diet

**Figure 3:**
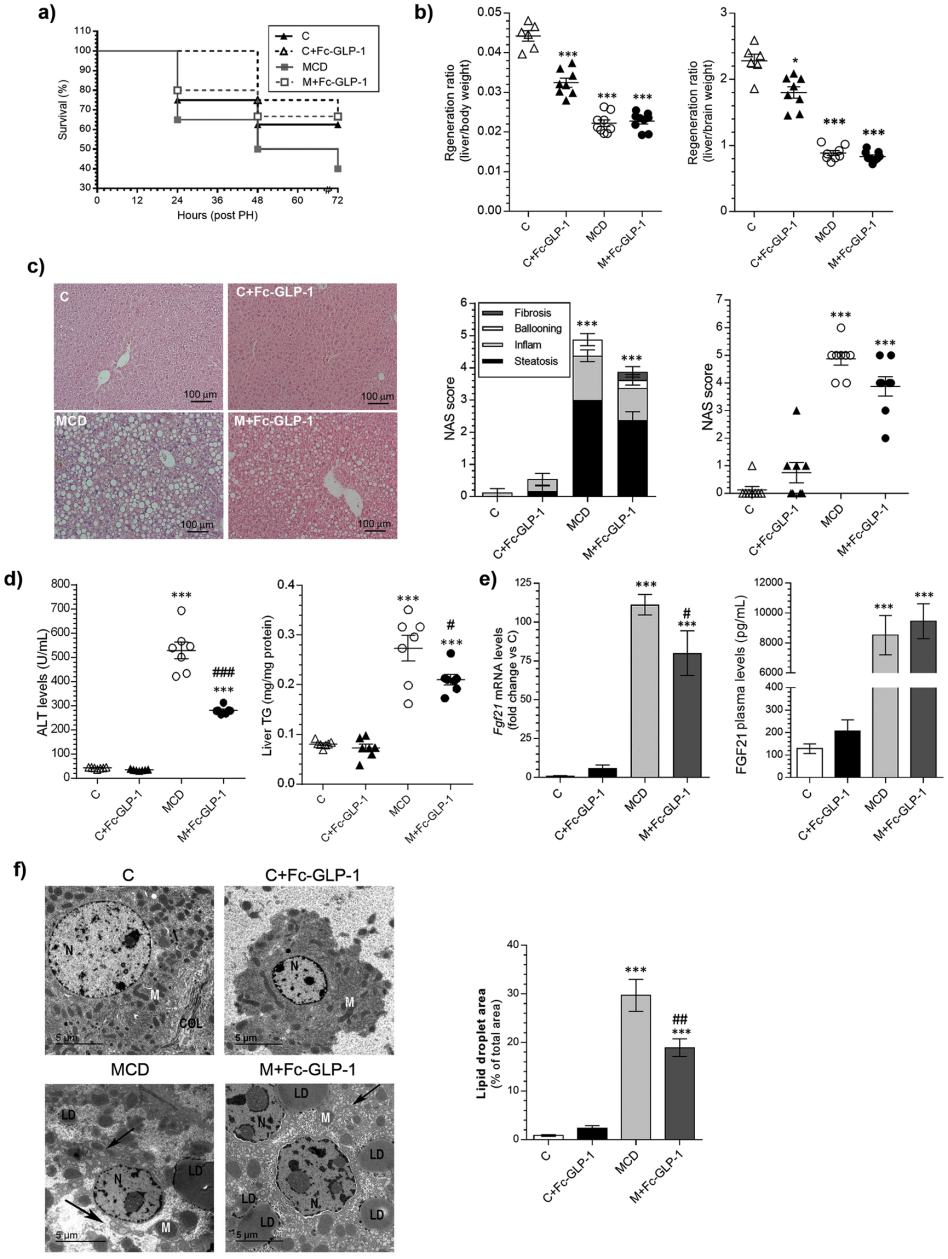
Effects of the Fc-GLP-1 treatment on PLF as evaluated by PH-induced hepatic regeneration in mice fed chow (C) or MCD diet. (**a**) Survival ratio after PH in different animal groups (n = 16 mice in C, 12 mice in C+Fc-GLP-1, 20 mice in MCD and 15 mice in M+Fc-GLP-1). (**b**) Regeneration ratio analyzed 2 weeks after PH corrected by body weight (left) or brain weight (right) (*p = *0*.*033*) (n = 6 mice in C, 8 mice in C + Fc-GLP-1, 9 mice in MCD and 9 mice in M + Fc-GLP-1). (**c**) Representative H&E images from liver sections of the different groups analyzed 2 weeks after PH and histopatologycal evaluation of the NAS score (n = 8 mice/group). (**d**) Effects of Fc-GLP-1 on ALT levels and liver TG content (^#^*p* = *0*.*0155*) (n = 7 mice/group). (**e**) Hepatic *Fgf21* mRNA levels (n = 6 mice/group; ^#^*p* = *0*.*0358*) and plasma FGF21 (n = 7 mice/group). (**f**) Representative TEM images of liver sections from different animal groups analyzed 2 weeks after PH showing lipid droplets (LD), mitochondrion (M), nucleus (N), dilated ER (black arrow) and quantification of the area of LD (4 random fields per mice; n = 3 mice/group; ^##^*p* = *0*.*0038*). ***p < *0*.*001 vs C group*; ^###^*p* < *0*.*001 vs MCD group according to one-way ANOVA with post-hoc Bonferroni test*.

We next investigated the effect of Fc-GLP-1 on PLF by evaluating liver regeneration after PH. In mice on MCD diet the treatment with Fc-GLP-1 significantly improved PLF. In fact, only 38% of mice of this group survived at 72 h post-PH whereas survival rate reached 66.7% in the M+Fc-GLP-1 group (Fig. 3a). In addition, mice on MCD diet presented decreased liver mass recovery at 2 weeks post-PH and this reduction was also evident in the M+Fc-GLP-1 group (Fig. 3b). Liver mass recovery was slightly, but statistically significantly, reduced in mice on a chow diet receiving Fc-GLP-1. Importantly, in both groups receiving treatment with Fc-GLP-1 the drug was detected in plasma 48 h after the last injection (Supplementary Fig. 2), excluding the possibility of reduced circulating drug levels after PH.

The histological analysis of liver samples collected at 2 weeks post-PH revealed that the surviving mice of the MCD group presented elevated global NAS score, plasma ALT, intrahepatic TG and increased hepatic *Fgf21* mRNA and serum levels of FGF21 (Fig. 3c–e). In addition, serum insulin was decreased in mice fed a MCD diet regardless of the treatment with Fc-GLP1 (Supplementary Fig. 3). However, contrary to the beneficial effects of Fc-GLP-1 in the reversion of NASH-like features before PH (Fig. 1a,b), reduced efficacy of Fc-GLP-1 on liver disease was evident in the M + Fc-GLP-1 mice at 2 weeks post-PH (Fig. 3c–e), although the microarray analysis revealed up-regulation and enrichment in gene sets related to NASH in MCD versus M + Fc-GLP-1 group (Supplementary Table 3). EM images showed rounded mitochondria, large lipid droplets and ER dilatation in both MCD and M+Fc-GLP-1 groups (Fig. 3f). Moreover, intrahepatic glycogen was significantly increased only in the C+Fc-GLP-1 group (Supplementary Fig. 5). Similar results regarding PLF, evolution of NASH-like features and liver mass recovery were found using liraglutide, a different GLP-1R agonist (Supplementary Fig. 6).

### Analysis of immune cells and HSC markers in livers from Fc-GLP-1 treated mice after PH

**Figure 4:**
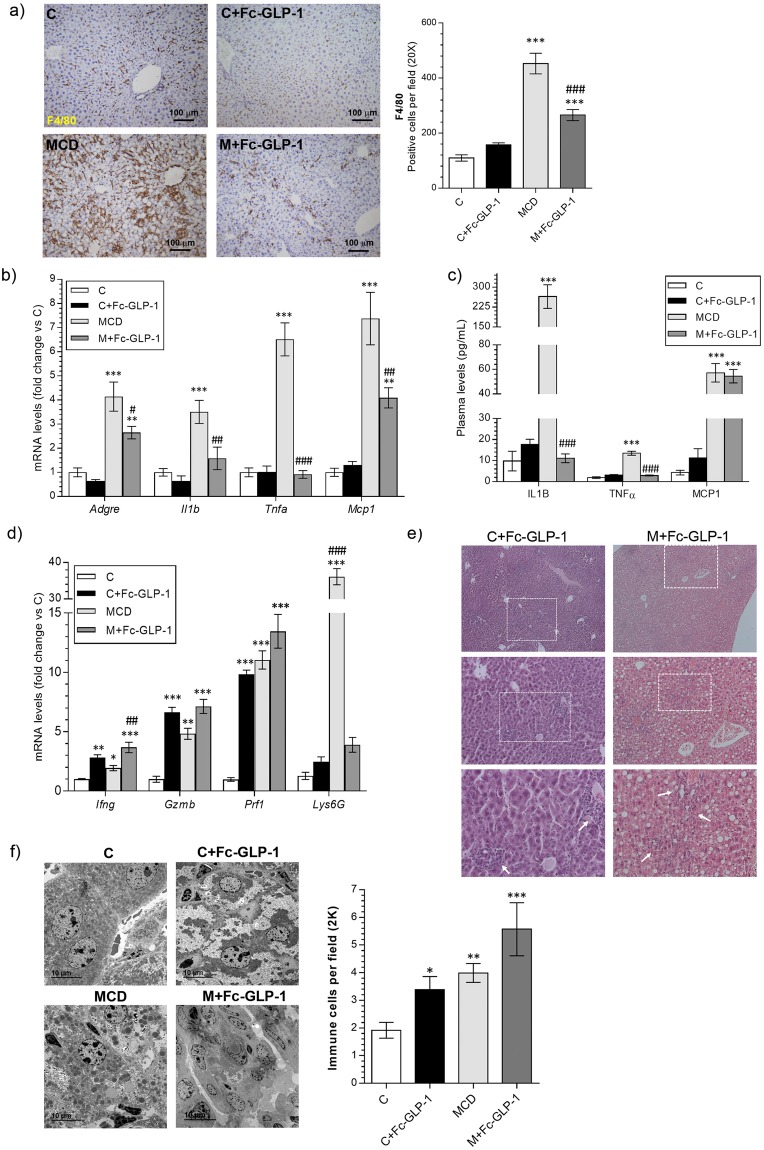
Analysis of immune cells markers in livers from Fc-GLP-1 treated mice after PH. (**a**) Representative images and quantification of F4/80 immunohistochemistry analyzed 2 weeks after PH (3 random fields per mice; n = 4 mice/group). (**b**) Hepatic mRNA levels of proinflammatory cytokines (n = 6 mice/group). (**c**) Plasma levels of proinflammatory cytokines analyzed 2 weeks after PH (n = 6 mice/group). (**d**) mRNA levels of cytotoxic lymphocytes and neutrophils related genes after PH (n = 6 mice/group). Representative H&E (n = 8 mice/group) (**e**) and TEM (**f**) images of Fc-GLP-1 treated animals showing infiltration of non-parenchymal immune cells and quantification of the immune cells in the TEM (4 random fields per mice; n = 3 mice/group; *p = *0*.*0157*; **p = *0*.*0038*). *p < *0*.*05*, **p < *0*.*01*, ***p < *0*.*001 vs C group*; ^#^*p* < *0*.*05*, ^##^*p* < *0*.*01*, ^###^*p* < *0*.*001 vs MCD group according to one-way ANOVA with post-hoc Bonferroni test*.

We next analyzed the evolution of several inflammatory markers during the hepatic regenerative process. As shown in Fig. 4a, F4/80 immunostaining confirmed persistent hepatic inflammation in the MCD group at 2 weeks post-PH that was also present, although to a lesser extent, in M+Fc-GLP-1 mice. Analysis of inflammatory cytokines in liver and plasma showed reductions in IL1β and TNFα in mice receiving MCD diet and Fc-GLP-1 (Fig. 4b,c). However, hepatic *Mcp1* mRNA levels and circulating MCP1 remained highly elevated in both MCD and M+Fc-GLP-1 groups. Additional analysis of natural killer (NK) and cytotoxic T lymphocytes (CD8+) revealed that in the C+Fc-GLP-1 group *Ifng*, *Gzmb* and *Prf1* mRNAs were significantly increased compared with the chow group (Fig. 4d), and these markers were also upregulated in the M+Fc-GLP-1 group. Indeed, in both C+Fc-GLP-1 and M+Fc-GLP-1 groups, immune cells were visualized in liver sections (Fig. 4e) and quantified in the TEM images (Fig. 4f).

**Figure 5:**
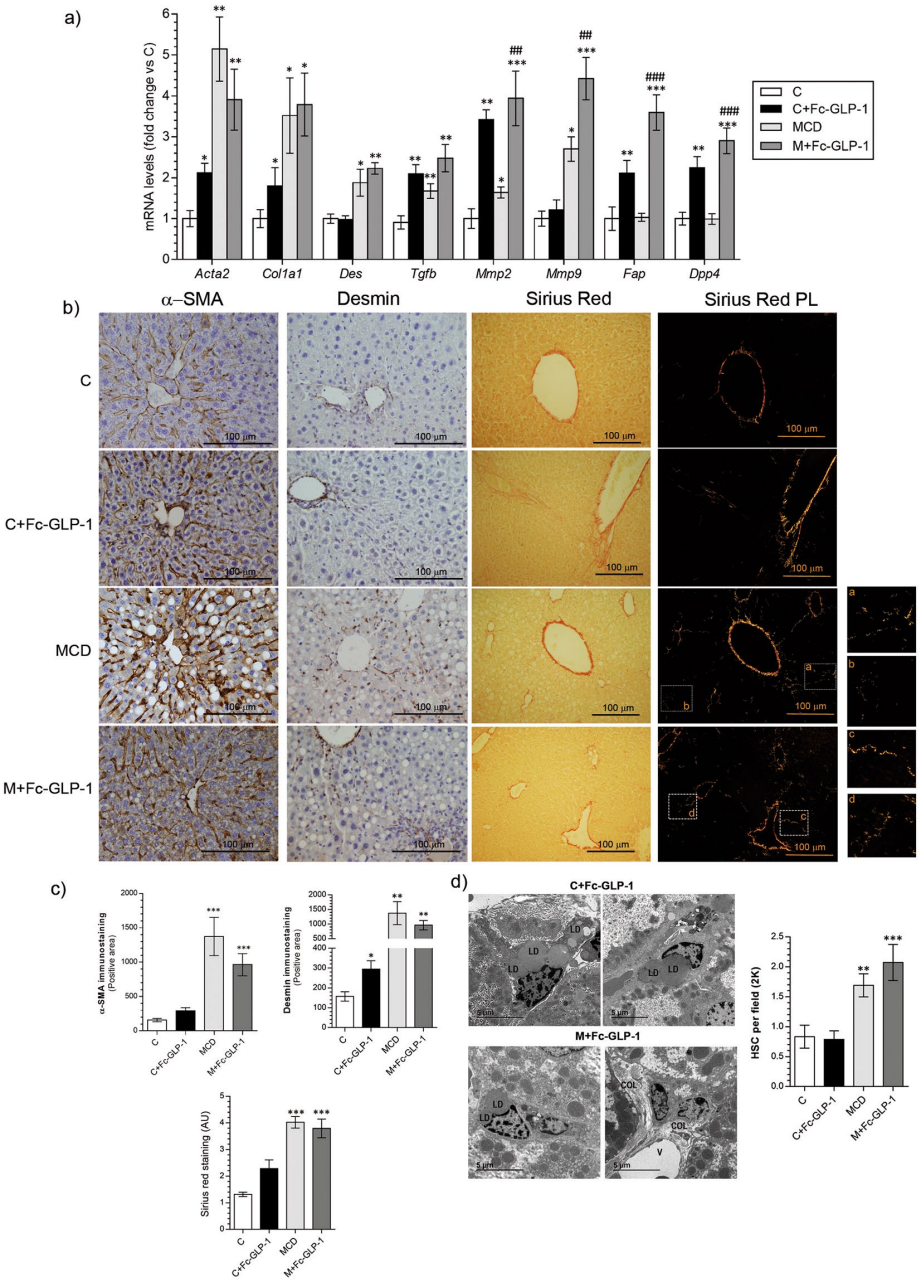
Analysis of markers of HSC activation in livers from Fc-GLP-1 treated mice 2 weeks after PH. (**a**) Hepatic mRNA levels of HSC related genes analyzed 2 weeks after PH (n = 6 mice/group). (**b**,**c**) Representative images and quantification of α-SMA and desmin immunohistochemistry (n = 4 mice/group; 3 random fields per mice; *p = *0*.*018* (C+Fc-GLP-1); **p = *0.00014* (MCD) and **p = *0.00019* (M+Fc-GLP-1) and Sirius Red staining (2 fields per mice; n = 4 mice/group) at 2 weeks after PH. (**d**) Representative TEM images of livers from M+Fc-GLP-1 mice showing hepatic stellate cells (HSC) containing lipid droplets (LD) and collagen fibers (COL) surrounding vein (V) and quantification of HSC (4 random fields per mice; n = 3 mice/group). *p < *0*.*05*, **p < *0*.*01*, ***p < *0*.*001 vs C group*; ^##^*p* < *0*.*01*, ^###^*p* < *0*.*001 vs MCD group according to one-way ANOVA with post-hoc Bonferroni test*.

It has been postulated that HSC are a major source of extracellular matrix (ECM) proteins during liver regeneration^25^. Also, activated HSC secrete high levels of MCP1^26^. Based on the elevations in *Mcp1* mRNA detected at 2 weeks post-PH in livers of mice receiving Fc-GLP-1 (Fig. 4b,c), we analyzed key markers of HSC activation. Treatment with Fc-GLP-1 in mice fed a chow diet significantly increased *Acta2* (encoding α-SMA), *Col1a1*, *Tgfb* and *Mmp2* mRNAs (Fig. 5a). HSC markers were elevated in livers of MCD mice after PH, as reported^27^, and remained elevated in the M + Fc-GLP-1 group; this effect being particularly evident with *Mmp2* and *Mmp9* expression. Likewise, increased expression of *Dpp4* and *Fap* was observed in the two groups of Fc-GLP-1-treated mice. Anti-α-SMA and anti-desmin immunostaining verified these data in MCD diet-fed mice (Fig. 5b,c). Also, positive Sirius Red staining was observed in the parenchyma of livers from MCD and M + Fc-GLP-1 groups. In addition, in those mice (MCD and M + Fc-GLP-1) TEM images showed an accumulation of HSC in the Disse Space (Fig. 5d and Supplementary Fig. 7).

### Changes in gene expression related to inflammation and activation of HSC in Fc-GLP-1 treated mice after PH

**Figure 6:**
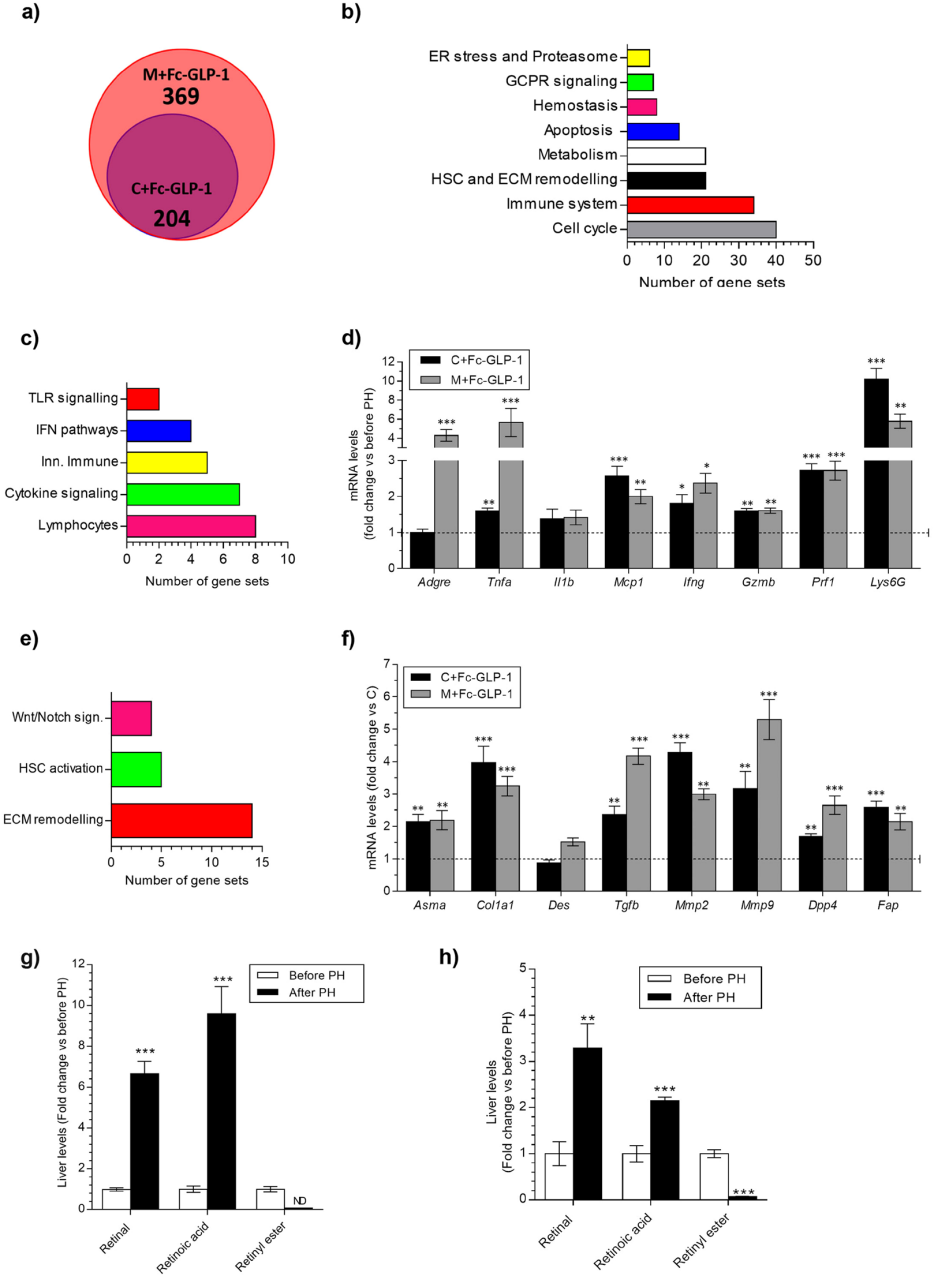
Comparison of the microarray data of livers from Fc-GLP-1 treated mice before and after PH by GSEA software. (**a**) Venn diagram of gene sets up-regulated in C+Fc-GLP-1 and M+Fc-GLP-1 groups 2 weeks after PH versus values of each individual group analyzed in liver samples removed in the hepatectomy (n = 4 mice/group). (**b**) Gene sets commonly up-regulated in both C+Fc-GLP-1 and M+Fc-GLP-1 groups versus values analyzed in liver samples removed in the hepatectomy. (**c**) Principal inflammatory gene sets up-regulated in Fc-GLP-1 treated animals 2 weeks post-PH versus values obtained in liver samples removed in the hepatectomy (n = 4 mice/group). (**d**) mRNA levels of pro-inflammatory cytokines, cytotoxic lymphocytes and neutrophils related genes analyzed 2 weeks after PH in comparison with values of each individual group measured in liver samples removed in the hepatectomy (n = 6 mice/group). (**e**) Principal HSC-related gene sets up-regulated in Fc-GLP-1 treated animals as indicated above (n = 4 mice/group). (**f**) mRNA levels of genes related to HSC activation in Fc-GLP-1- treated animals as indicated above (n = 6 mice/group). Fold change of hepatic levels of metabolites related to activation of HSC in C+ Fc-GLP-1 (**g**) and M+ Fc-GLP-1 (**h**) under conditions indicated above (n = 6 mice/group). *p < *0*.*05*, **p < *0*.*01*, ***p < *0*.*001 vs each group before PH according to one-way ANOVA with post-hoc Bonferroni test*.

We further explored the evolution of inflammation and HSC related gene expression in livers from the 2 groups of mice treated with Fc-GLP-1 (fed chow or MCD diet) after PH. Microarray analysis revealed that in mice receiving treatment with Fc-GLP-1 numerous gene sets were modulated by the regeneration process (Supplementary Tables 4 and 5 and Supplementary Fig. 8). In particular, the Venn diagram (Fig. 6a) shows a total of 204 gene sets up-regulated in livers of mice fed a chow diet administered Fc-GLP-1 (C+Fc-GLP-1) at 2 weeks post-PH compared to the values of liver samples of this experimental group removed during the PH surgery. Likewise, a total of 573 gene sets were up-regulated in M+Fc-GLP-1 mice under the same conditions. We also found that 204 gene sets up-regulated in the C+Fc-GLP-1 group after PH were also up-regulated in the M+Fc-GLP-1 group. Among them, 34 gene sets were related to inflammation, 21 to metabolism and 21 to HSC (Fig. 6b and Supplementary Table 5). Figure 6c,e detail the inflammatory and HSC related gene sets commonly modulated by Fc-GLP-1 treatment after PH. Validation of inflammatory genes revealed that *Tnfa*, *Mcp1*, *Ifng*, *Gzmb*, *Prf1* and *Ly6G* mRNAs were up-regulated in C+Fc-GLP-1 mice at 2 weeks post-PH compared with the values obtained in liver samples removed during the PH surgery (Fig. 6d), suggesting that continuation with the treatment with Fc-GLP-1 after PH, thereby during the liver regeneration process, promoted the activation of different immune cell populations such as monocytes, NK cells, cytotoxic T lymphocytes and neutrophils. A similar profile was found in livers of the M+Fc-GLP-1 group with exacerbated increases in A*dgre1* (encoding F4/80) and *Tnfa* mRNAs after PH. Next, we performed a similar analysis with markers of HSC activation by comparing the values of liver samples collected 2 weeks post-PH with the values of the liver samples removed during the PH surgery. In both groups of Fc-GLP-1 treated mice, *Acta2*, *Col1a1*, *Tgfb*, *Mmp2*, *Mmp9*, *Dpp4* and *Fap* mRNAs were up-regulated, indicating enhanced HSC activation by Fc-GLP-1 administration after PH during the regenerative process (Fig. 6f). We also performed metabolomics focusing on HSC metabolism. As depicted in Fig. 6g (C + Fc-GLP-1 group) and 6h (M + Fc-GLP-1 group), at 2 weeks post-PH, levels of retinyl ester were significantly decreased in livers of mice receiving Fc-GLP-1 in parallel to an increase in its derived metabolites retinal and retinoic acid.

### Fc-GLP-1 improved liver PLF, but did not alleviate NAFLD after PH in diet-induced obese (DIO) mice

**Figure 7:**
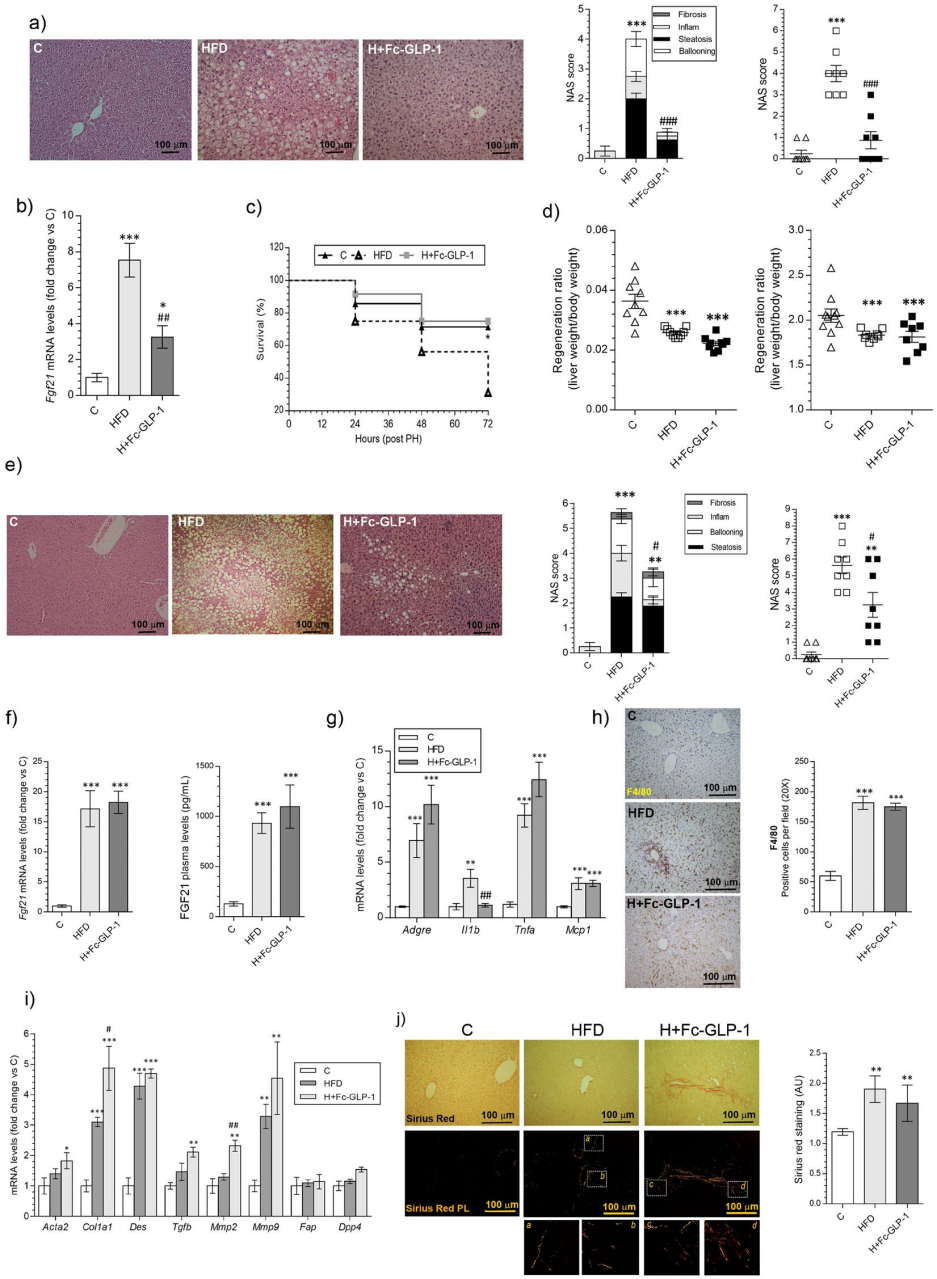
Fc-GLP-1 improved liver PLF, but did not alleviate NAFLD after PH in DIO mice. (**a**) Representative images of H&E from mice fed a chow diet (C) or high fat diet (HFD) untreated or treated with Fc-GLP-1 (H + Fc-GLP-1) (left panel) and evaluation of the NAS score (right panel) before PH (n = 9 mice/group). (**b**) Effect of Fc-GLP-1 on *Fgf21* mRNA levels before PH (n = 8 mice/group; *p = *0*.*028*, ^##^*p* = *0*.*007*). (**c**) Survival ratio after PH in the different animal groups (n = 14 mice in C, 18 mice in HFD and 12 mice in H + Fc-GLP-1; *p = *0*.*0375*). (**d**) Regeneration ratio determined 2 weeks after PH (n = 8 mice/group). (**e**) Representative images of H&E from mice fed a chow diet (C) or HFD untreated or treated with Fc-GLP-1 (H + Fc-GLP-1) 2 weeks after PH and histopathologycal evaluation of the NAS score (n = 8 mice; **p = *0*.*0019*, ^#^*p* = *0*.*0135*). (**f**) Effect of Fc- GLP-1 on *Fgf21* mRNA levels (n = 6 mice/group) and plasma FGF21 (n = 7 mice/group) analyzed at 2 weeks after PH. mRNA levels of genes related to inflammation (**g**) and HSC activation (**i**) (n = 6 mice/group). Representative images and quantification of F4/80 immunohistochemistry (3 random fields per mice; n = 4 mice/group) (**h**) and Sirius Red staining (**j**) at 2 weeks after PH (2 random fields per mice; n = 4 mice/group). *p < *0*.*05*, **p < *0*.*01*, ***p < *0*.*001 vs C group*; ^#^*p* < *0*.*05*, ^##^*p* < *0*.*01*, ^###^*p* < *0*.*001 vs MCD group according to one-way ANOVA with post-hoc Bonferroni test*.

The effect of Fc-GLP-1 in NAFLD and liver mass recovery was assessed in DIO mice (Supplementary Fig. 1B). Obese mice fed a HFD for 10 weeks were injected Fc-GLP-1 for 3 additional weeks. As expected, Fc-GLP-1 decreased body weight (Supplementary Fig. 9) and, as occurred in the MCD model, improved liver histology, reduced the NAS score and decreased hepatic *Fgf21* mRNA levels (Fig. 7a,b). After PH, Fc-GLP-1 doubled survival in mice fed a HFD (Fig. 7c), but did not enhance liver mass recovery (Fig. 7d). In agreement with data in non-obese MCD model, histopathologycal evidence of NAFLD was found 2 weeks after PH in DIO mice receiving Fc-GLP-1 (Fig. 7e). Likewise, we did not find differences in hepatic *Fgf21* mRNA and FGF21 plasma levels between the HFD and H+Fc-GLP-1 groups 2 weeks after PH (Fig. 7f). Inflammation, assessed by the analysis of mRNA levels of proinflammatory cytokines (Fig. 7g) and F4/80 immunostainning (Fig. 7h), together with activated HSC-related gene expression (Fig. 7i) and Sirius Red staining (Fig. 7j) was detected in H+Fc-GLP-1 mice 2 weeks after PH to a similar extend as in the HFD group. Altogether, these data in DIO mice reinforce the data from the MCD model regarding the lack of effect of Fc-GLP-1 in alleviating NAFLD in regerating livers.

## Discussion

During the last few years preclinical studies have demonstrated the efficacy of GLP-1R agonists in alleviating NASH^13,17,28–31^. GLP-1’s, therefore, represent a new therapeutic opportunity in tackling the global epidemic of this chronic liver disease. Here, we used two dietary intervention models in mice: MCD diet to induce NASH-like features in the context of leaness^32^ and HFD which induces obesity-associated fatty liver and metabolic disturbances, to demonstrate that Fc-GLP-1 alleviated NAFLD and improved survival following PH. These two divergent models of liver dysfunction were selected to explore the broad potential application and associated mechanisms of GLP-1 based therapies on various hallmark characteristics of NAFLD, as well as on hepatic regeneration following PH in differing metabolic contexts.

Microarray analysis of livers from MCD diet-fed mice identified different gene sets modulated by the treatment with Fc-GLP-1 for 2 weeks. Among them, we validated changes in inflammatory genes such as *Tnfa*, *Il1b*, *Adgre* (encoding F4/80) and *Mcp1*, all of them decreased by Fc-GLP1 that was also reflected by reductions in F4/80 immunostaining in liver sections. These data highlight the efficacy of Fc-GLP-1 in targeting macrophage infiltration. Fc-GLP-1 administration was also associated with a reduction in the activation of HSC in the MCD model. However, while *Col1a1* and *Tgfb* mRNAs were decreased with Fc-GLP-1 treatment, other markers such as *desmin*, *Mmp2*, *Mmp9* and *αSma* remained elevated. This result is intriguing but was reinforced by the visualization of HSC in the Disse space in livers from mice fed a MCD diet receiving treatment with Fc-GLP-1. Fc-GLP-1 also improved the fatty liver phenotype in DIO mice, confirming the efficacy of this GLP-1R agonist in obese and non-obese hepatic dysfunction linked to NAFLD.

It is noteworthy to mention that the direct effect of GLP-1R agonists in hepatic cells has been a subject of controversy. On one hand, several studies found expression of GLP-1R in hepatocytes^11,31,33,34^, whereas others indicated the opposite^35–38^. Tomas *et al*.^38^ demonstrated that GLP-1 9–36 suppress glucose production in isolated mouse hepatocytes. On the other hand, an *in vivo* study with the exenatide analog AC3174 showed reduced hepatic lipids in wild-type mice fed a high *trans*-fat diet, but not in mice lacking GLP-1R^15^. Whether the effects of Fc-GLP-1 in MCD diet- and HFD-fed mice are due to direct or indirect actions of GLP-1 on liver are not clear from these studies and require further investigation.

A relevant finding in our study was the improvement of post-PH liver failure in all the groups of mice administered Fc-GLP-1 regardless of the dietary intervention. This benefit was manifested by reduced mortality after PH. This issue might be of clinical interest since PLF is a major source of morbidity and mortality in patients undergoing liver resection with an incidence between 0.7 and 34% depending on the severity of the surgery^39^. More research will be needed to decipher the molecular basis for this effect; a possible explanation could be related to a hepatoprotective role of GLP-1. GLP-1 protects HepG2 cells against high glucose-induced apoptosis by reducing the pro-apoptotic genes Bak and Bax^40^. In the same study, elevations in PGC-1α were found upon GLP-1 treatment of hepatic cells. As PGC-1α is a master gene in the regulation of hepatic metabolism, both survival and improved metabolic function of liver cells might be crucial effects of Fc-GLP-1 to ensure a better recovery from PH. Alternatively, systemic effects of GLP-1 regarding whole-body metabolism could participate in this beneficial effect. Thus, administration of a GLP-1R agonist to patients undergoing liver resection in combination with the management principles recommended by the AASLD might synergize in the prevention of PLF occurrence.

Our recent work on the benefits of administration of G49, a dual GLP-1R/GCGR agonist, in liver regeneration during NASH^27^ prompted us to decipher whether this effect could be recapitulated with a selective GLP-1R agonist. A first unexpected finding was the manifested simptoms of NAFLD at 2 weeks post-PH in livers from mice treated with Fc-GLP-1 fed a MCD diet or HFD despite the marked improvements in liver histology already detected in those livers at the moment of the liver resection. Notably, sustained levels of Fc-GLP-1 in plasma were found in hepatectomized animals excluding a drop of drug levels as a potential explanation for the NAFLD detected after PH. At present, we cannot provide a rational explanation to the opposite effects of Fc-GLP-1 in PLF and liver mass recovery that might be related to the complexity of the regenerative process of the liver. During hepatic regeneration, increased activity of urokinase plasminogen activator (uPA) triggers extracellular matrix (ECM) remodeling involving cascades of metalloproteinase activation which lead to the release of active HGF in the ambient environment of hepatocytes and in circulation and this is followed by a plethora of events to restore the architecture of the liver^41,42^. In this scenario, the intrahepatic environment during regeneration, markedly different from that of the quiescent liver, might impact on the efficacy of the GLP-1R agonist in ameliorating NAFLD. Indeed, microarray analysis showed 7 gene sets related to ECM including ECM receptor interaction, regulation of actin cytoskeleton and TGFβ signaling up-regulated in M + Fc-GLP-1 at 2 weeks post- PH *versus* values of the same group measured in liver samples removed in the liver resection (Supplementary Fig. 10), suggesting a possible effect of Fc-GLP-1 in ECM remodeling after PH. Interestingly, in pancreatic tissue of diabetic rats treated subcutaneously with a GLP-1R agonist for 10 weeks, αSMA, collagen III, MMP2 and MMP9 were increased^43^. In this study it was suggested the existence of pancreatic stellate cells within the pancreatic tissue that may be highly sensitive to drugs such as GLP-1R agonists and more susceptible to be activated to cause chronic inflammatory disease. In fact, genes related to adaptive immune system were up- regulated in M + Fc-GLP-1 mice post-PH (Supplementary Fig. 11) which might reflect an immune response to face apoptosis triggered by liver resection. Therefore, changes in ECM and adaptive immunity and probably additional processes associated with the restoration of liver mass after PH might confer a less adequate environment for Fc-GLP- 1 action. Altogether, these data suggest that Fc-GLP-1 exhibits reduced efficacy in the context of NAFLD plus liver resection, an observation with a potential relevance in the clinical settings. Thus, more research will be needed to understand whether this complex issue can be translated to human patients with this chronic liver disease.

A rationale to explain the opposite effects of G49, a dual GLP-1R/GCGR agonist^27^, and Fc-GLP-1 in liver mass recovery may be due to their different roles in glucose metabolism. Coordinated glucose metabolism is a prerequisite for normal liver regeneration. Following PH, the reduction in liver mass leads to hormonal changes that shift glucose metabolism towards glycogenolysis and gluconeogenesis in the remnant liver^44^ and promote glucose utilization towards the pentose phosphate cycle to provide NADPH for lipid and DNA synthesis^45^. Thus, the action of G49 via GCGR may result in enhanced hepatic glucose mobilization providing appropriate metabolic intermediaries necessary to drive liver regeneration following PH^27^. Regarding the effect of GLP-1 alone in intrahepatic glucose content, exendin-4 was reported to increase glycogen synthesis in murine hepatocytes^46^, likely contributing to the replenishment of hepatic glucose stores. However, we did not find differences in glycogen stores among mice fed a MCD diet with or without Fc-GLP-1 treatment, excluding differences in hepatic glycogen as the responsible for the lack of effect of Fc-GLP-1 in hepatic regeneration in the context of NASH-like features. In addition, as GLP-1 has been shown to reduce gluconeogenesis^47^ which, as stated above, is necessary to provide fuel for liver regeneration, this effect might limit its efficacy for the recovery of the liver mass after PH. Interestingly, liver mass recovery was also decreased in mice with normal liver treated with Fc-GLP-1, similar to that reported in rats treated with liraglutide following PH^48^. It was suggested that this effect was due to reductions in TAG, since lipids are fuel for liver mass recovery. Thus, it is possible that the decreased TAG content in livers from mice with normal liver treated with Fc-GLP-1 for 2 weeks before PH might limit the lipid availability required for regeneration.

In conclusion, our results shed light into the differential effects of a long-acting GLP-1R agonist in improving NAFLD and PLF in the context of obesity and leaness, but decreasing liver mass recovery after PH. Since the current epidemic of end-stage liver diseases, including NAFLD, limits liver transplantation, more preclinical studies are needed to test the efficacy of drugs such as G49 and to identify new therapeutic targets.

## Materials and Methods

### Animals, diets, PH and treatments

8 week-old male C57Bl/6 mice were fed a chow diet or MCD diet (TD-90262 Harlan-Tecklad, Indianapolis, USA) for one week, at which time liver damage is already observed^27^. Mice were subsequently divided into 4 groups as follows: mice that continued receiving chow diet for 2 additional weeks (C), mice fed a chow diet and subcutaneously (s.c.) injected Fc-GLP-1 (GLP-1(7–36)NH2 sequence HGEGT FTSDV SSYLE GQAAK EFIAW LVKGG-Fc at 10 nmol/kg every two days (C + Fc-GLP-1), mice that continued with MCD diet (MCD) and mice fed a MCD diet and injected Fc-GLP-1 (M + Fc-GLP-1). Both C and MCD groups were injected with vehicle (saline). Then, mice were subjected to 70% PH as described^49^, after that animals were maintained on similar diet and Fc-GLP-1 treatment. Mice were sacrificed 15 days after PH and livers were removed, weighed, and normalized to body or brain weight. Another cohort of mice was fed a HFD (60% of calories from fat, TD-06414) for 10 weeks, injected Fc-GLP1 (H + Fc-GLP-1) or vehicle (HFD) for 3 additional weeks and subjected to 70% PH. Animals were subsequently maintained on the same diet and treatment with Fc-GLP- 1 and sacrificed 2 weeks post-PH. Animal experimentation was approved by the Ethic Committee at Spanish Researh National Council (CSIC. Spain) and was conducted accordingly to accepted guidelines for animal care of Comunidad de Madrid (Spain).

### Synthesis of Fc-GLP-1

GLP-1 analogue G8, E22,G36-GLP-1 (referred as Fc-GLP-1) was genetically fused to the N-terminus of a human IgG4 Fc via a (G4S)3 flexible linker in a mammalian expression vector as previously described^50^. The GLP-1 Fc-fusion protein was transiently expressed in Chinese hamster ovary cells under serum-free conditions. Cleared culture supernatant was loaded directly onto MapSelect SuRe column equilibrated with PBS (pH 7.2). GLP-1 Fc-fusion was eluted with a gradient (15 column volumes) 50 mM sodium acetate, pH 3.65. Pooled fractions were equilibrated to 25 mM Histidine, 7% Sucrose, 0.02% polysorbate 80, pH 6.0 by tangential flow filtration using a 30 kDa cut-off filter. The purified GLP-1 Fc-fusion contained at least 98.6% monomer when characterized by HPLC-SEC analysis. Intact mass of the constructs was confirmed by mass spectrometry and concentration assessed (absorption at 280 nm)^16^.

### Analysis of Fc-GLP-1 levels in mouse plasma

The concentration of Fc-GLP-1 present in mouse plasma samples was estimated from its agonist activity against the human GLP-1R expressing CHO cell line. Reference Fc-GLP-1 peptide was spiked into naïve mouse plasma at a known concentration to generate a standard curve to determine the amount of active peptide in each related plasma test sample. All samples were serially diluted in assay buffer in duplicate and incubated with cells for 30 min at RT before detection with the cAMP dynamic 2 HTRF assay (Cisbio assays, Bedford, MA) as described previously^51^. Plasma test samples were plotted using the same top concentration as the equivalent reference. EC50 values obtained using this method were then used to calculate: sample ratio = sample EC50/EC50 reference peptide-spiked plasma sample and estimated concentration = Known top concentration of peptide- spiked plasma sample/sample ratio.

### Serum cytokine detection assay

Serum samples were collected and immediately frozen at −80 °C. TNFα, IL6, IL1β and MCP-1 were determined using Luminex 100 IS (Merck Millipore, Darmstadt, Germany).

### Determination of alanine aminotransferase (ALT) levels

ALT activity was determined in plasma using Reflotron strips (Roche Diagnostics, Barcelona, Spain), according to the manufacturer’s instructions.

### Plasma levels of insulin and FGF21

Plasma insulin was analyzed by ELISA (Mercodia Insulin ELISA, Ref. 10–1113) and plasma FGF21 was detected with the FGF21 ELISA (Biovendor, Ref. RD291108200R).

### Liver histology

Paraffin-embedded liver biopsy sections were stained with Hematoxylin & Eosin and evaluated by a single blinded hepatopathologist. Steatosis was assessed as outlined by Kleiner *et al*.^52^ grading percentage involvement by steatotic hepatocytes as follows: grade 0, <5%; grade 1, 5–33%; grade 2, >33–66%; and grade 3, >66%. In addition, Brunt’s histological scoring system^53^ was used to evaluate the degree of hepatocellular ballooning and lobular inflammation (grade of activity) as well as the stage of fibrosis. Minimal criteria for the histological diagnosis of definite NASH included the combined presence of grade 1 steatosis, hepatocyte ballooning, and lobular inflammation with or without fibrosis.

### Transmission electron microscopy (TEM) and immunohistochemistry

Liver samples were fixed in glutaraldehyde (2%) plus 4% *p-*formaldehyde for 60 min. Samples were post-fixed in 1% osmium tetroxide for 60 min at 25 °C, stained with uranyl acetate (5 mg/mL) for 1 h at 25 °C, dehydrated in acetone and embedded in Epon 812 (EMbed 812; Electron Microscopy Science, Hatfield, PA). Ultrathin sections, unstained or post- stained with uranyl acetate and lead hydroxide were examined under a Morgagni 268D TE microscope (FEI, Hillsboro, OR) equipped with a Mega View II charge-coupled device camera (SIS, Soft Imaging System GmbH, Munster, Germany) and analyzed with AnalySIS software (SIS). Liver immunohistochemistry was performed as previously described^54^. The antibodies used were anti-F4/80 (MCA497, Serotec) and anti-αSMA (A2547, Sigma). Sirius staining was performed as previously described^27^.

### Determination of intrahepatic triglyceride (TG) content

Hepatic lipids were extracted as described^55^. After purification, lipids were re-suspended in isopropyl alcohol and TG were analyzed with a colorimetric kit (Biosystems, Barcelona, Spain).

### Determination of intrahepatic glucose and glycogen

Glycogen content was quantified as previously described^56^ with modifications. Briefly, frozen tissue samples were dissolved in 30% KOH by immersion in boiling water for 1 h. Glycogen was precipitated by the addition of ice-cold absolute ethanol and storing the samples overnight at −20 °C in 2 mL tubes. The resulting pellet from a 1,000 × g centrifugation was re-dissolved in H_2_O and precipitated again with ice-cold ethanol. The purified glycogen was then hydrolyzed to glucose by amyloglucosidase and the liberated glucose was quantified using the glucose oxidase enzymatic method (Biosystems, Barcelona, Spain). Free intrahepatic glucose was analyzed in samples before amyloglucosidase treatment.

### Quantitative real-time PCR analysis

Total RNA was extracted with Trizol® reagent (Invitrogen, Madrid, Spain) and reverse transcribed using a SuperScript™ III First- Strand Synthesis System for qPCR following the manufacturer’s protocol (Invitrogen). qPCR was performed with an ABI 7900 sequence detector. Primer-probe sets for mouse genes indicated in Figure Legends were purchased as predesigned TaqMan gene expression assays (Thermo Fisher Scientific, Madrid, Spain). For *Ifng*, *Gzmb*, *Prf1* and *Ly6G* mRNA determination primer sequences are available upon request.

### Microarray analysis

To discover differential liver gene expression, we performed microarray gene expression study by a SurePrint G3 mouse GE 8 × 60 K array (Agilent, Santa Clara, CA). Quality of the RNA samples was ensured in a Bioanalyzer 2100. The microarray comprises over 55,681 probe sets representing more than 39,430 well- substantiated mouse genes. Processing, normalization and differential expression was performed using limma Bioconductor package^57^. Enrichment of gene sets of interest were identified by Kyoto Encyclopedia of Genes and Genomes (KEGG) and REACTOME collections of MSigDB Molecular Signatures Database (MSigDB) by Gene Set Enrichment Analysis (GSEA) software (Broad Institute, USA) as described by Mootha *et al*.^58^. Raw and normalized microarray data have been uploaded GEO database (accession number GSE119725).

### Metabolomic analysis

A detailed description of the metabolomics analysis was previously described^27^.

### Statistical analysis

Data are reported as the mean ± SEM. Normal distribution was assessed by two different tests, Shapiro-Wilk and Kolmogorov-Smirnov. To determine the effects of diet or Fc-GLP-1 treatment, one-way ANOVA or Kruskal-Wallis followed by a Bonferroni or Dunn’s test, respectively, was carried out. The *p* values presented in figures and tables corresponded to post hoc test. For the survival studies, statistical analysis of Kaplan-Meier curves was performed with a Log-rank (Mantel- Cox) Test. For the body weight curve in DIO mice, a Student’s *t*-test was carried out. All statistical analyses were performed using the GraphPad Prism 5.0 software (GraphPad Software Inc., San Diego, CA, USA) and SPSS 15.0 software (SPSS, Inc., Chicago, IL, USA). Differences were considered statistically significant at *p* < 0.05. The statistical analysis for metabolomics and microarray data were previously described^27^.

## Electronic supplementary material

Supplementary Material
