# Supplementary material for: Differential Effects of a Glucagon-Like Peptide 1 Receptor Agonist in Non-Alcoholic Fatty Liver Disease and in Response to Hepatectomy
Source: Sci Rep. 2018 Nov 7;8:16461. doi: 10.1038/s41598-018-33949-z (PMC6220318; doi:10.1038/s41598-018-33949-z)
Supplement: Supplementary file 1 — Supplementary Material [file 41598_2018_33949_MOESM1_ESM.pdf]

SUPPLEMENTARY MATERIAL

DIFFERENTIAL EFFECTS OF A GLUCAGON-LIKE PEPTIDE 1 RECEPTOR AGONIST IN NON-ALCOHOLIC FATTY LIVER DISEASE AND IN RESPONSE TO HEPATECTOMY

M. Pilar Valdecantos[1,2#], Laura Ruiz[1,2], Virginia Pardo[1,2], Luis Castro-Sanchez[3], Carmelo García-Monzón[4,5], Borja Lanzón[6], Javier Rupérez[6], Coral Barbas[6], Jaqueline Naylor[7], James L. Trevaskis[8], Joseph Grimsby[8], Cristina M. Rondinone[8] and Ángela M. Valverde[1,2#].

[1]Instituto de Investigaciones Biomédicas Alberto Sols (Centro Mixto CSIC-UAM), Arturo Duperier 4, 28029 Madrid, Spain. [2]Centro de Investigación Biomédica en Red de Diabetes y Enfermedades Metabólicas Asociadas (CIBERdem), ISCIII, 28029 Madrid, Spain. [3]CONACyT-University of Colima, Colima 28045, Mexico. [4]Liver Research Unit, Santa Cristina University Hospital, Instituto de Investigación Sanitaria Princesa, Madrid 28009, Spain. [5]Centro de Investigación Biomédica en Red de Enfermedades Hepáticas y Digestivas (CIBERehd), ISCIII, 28029 Madrid, Spain. [6]Centre for Metabolomics and Bioanalysis (CEMBIO), Faculty of Pharmacy, Universidad San Pablo CEU, Campus Monteprincipe, Boadilla del Monte, 28668, Madrid, Spain. [7]MedImmune LTD, Cambridge, UK, [8]MedImmune LLC, Gaithersburg, MD 20878, USA.

Supplementary Table 1: Summary of microarray data performed in livers from mice fed chow or MCD diet treated with Fc-GLP-1.

	CHD vs C+Fc-GLP-1	MCD vs M+Fc-GLP-1
KEGG Up-regulated genes	74	105
KEGG Down-regulated genes	60	29
KEGG FDR<0.25 Up-regulated pathways	1	30
KEGG FDR<0.25 Down-regulated pathways		
REACTOME Up-regulated genes	115	238
REACTOME Down-regulated genes	176	53
REACTOME FDR<0.25 Up-regulated pathaways	1	79
REACTOME FDR<0.25 Down-regulated pathways	17	

Supplementary Table 2: Specific pathways related to the gene sets down-regulated in M+Fc-GLP-1-treated mice versus the MCD group.

Gene set	Pathways
Cell Cycle	K. Cell Cycle<br>K. Cytosolic DNA sensing pathway<br>K. DNA replication<br>R. Activation of ATR in response to replication stress<br>R. APC C CDC20 mediated degradation of mitotic proteins<br>R. APC C CDH1 mediated degradation of CDC20 and other APC C CDH1 targeted proteins in late mitosis-early G1<br>R. Assembly of the pre replicative complex<br>R. Cell Cycle<br>R. Cell Cycle checkpoints<br>R. Cell Cycle Mitotic<br>R. Cyclin E associated events during G1/S transition<br>R. DNA replication<br>R. DNA strand elongation<br>R. G alpha I signaling events<br>R. G1 S transition<br>R. G2 M checkpoints<br>R. Loss of NLP from mitotic centrosome<br>R. M G1 transition<br>R. Metabolism of mRNA<br>R. Mitotic G1 G1 S phases<br>R. Mitotic G2 G2 M phases<br>R. Mitotic M M G1 phases<br>R. Mitotic prometaphase<br>R. Nonsense mediated decay enhanced by the exon junction complex<br>R. ORC1 removal from chromatin<br>R. Regulation of mitotic cell cycle<br>R. RNA pol II pre transcription events<br>R. S phase<br>R. Synthesis of DNA
Inflammation	R. Immunoregulatory interactions between a lymphoid and a non-lymphoid cells<br>R. Chemokine receptors bind chemokines<br>R. Adaptive immune system<br>R. Immune system<br>R. IL-3,- 5 and GM-CSF signaling<br>R. Interferon gamma signaling<br>R. Interferon alpha, beta signaling<br>R. Innate immune system<br>R. Toll receptor cascades

	R. Interferon signaling<br>R. Activated TLR4 signaling<br>R. Cytokine signaling in immune system<br>K. Natural Killer cell-mediated cytotoxicity<br>K. Cytokine-cytokine receptor interaction<br>K. Chemokine signaling pathway<br>K. Leukocyte transendothelial migration<br>K. Toll like receptor signaling pathway<br>R. MHC CLASS II antigen presentation<br>R. Antigen processing cross presentation<br>R. CLASS I MHC mediated antigen processing presentation<br>R. Downstream signaling events of B cell receptor BCR<br>K. FC gamma R-mediated phagocytosis<br>K. B cell receptor signaling pathway<br>R. Signaling by the B cell receptor BCR<br>R. Co-stimulation by the CD28 family<br>R. TCR signaling<br>R. Activation of NF KAPPAB in B cells<br>K. NOD like receptor signaling pathway
Hemostasis and platelets	K. Complement and coagulation cascades<br>R. Cell surface interactions at the vascular wall<br>R. Hemostasis<br>R. Integrin-cell surface interactions<br>R. MYD88 mal cascade initiated on plasma membrane<br>R. PI metabolism<br>R. Platelet activation signaling and aggregation<br>R. Response to elevated platelet cytosolic Ca2+
ER Stress and proteasome	K. Proteasome<br>R. Activation of chaperone genes by XBP1S<br>R. Activation of genes by ATF4<br>R. PERK regulated gene expression<br>R. SCFSKP2-mediated degradation of P27 P21<br>R. Unfolded protein response
AA and purine metabolism	K. Amino sugar and nucleotide sugar metabolism<br>K. Purine metabolism<br>R. Amino acid transport across the plasma membrane<br>R. Asparagine N-linked glycosylation<br>R. Purine metabolism
Apoptosis and DNA damage	K. Base excision repair<br>K. RNA degradation<br>R. Amino acid transport across the plasma membrane<br>R. P53-dependent G1 DNA damage response<br>R. Regulation of apoptosis
Vesicles and Golgi	R. 3 UTR mediated translational regulation<br>R. SRP dependent cotranslational protein targeting to membrane<br>R. Translation

	R. Transport to the Golgi and subsequent modification
GPCR	R. Class A1 rhodopsin like receptors<br>R. GPVI-mediated activation cascade<br>R. Peptide ligand binding receptors
Glutathion metabolism	K. Glutathione metabolism<br>R. Glutathione conjugation
HSC	K. Retinol metabolism<br>R. Collagen formation

R; Reactome database. K; KEGG database.

Supplementary Table 3: Summary of the microarray analysis performed in livers from the different experimental groups at 2 weeks after partial hepatectomy

	CHD vs C+Fc-GLP-1	MCD vs M+Fc-GLP-1
KEGG Up-regulated genes	132	118
KEGG Down-regulated genes	2	16
KEGG FDR<0.25 Up-regulated pathways	43	25
KEGG FDR<0.25 Down-regulated pathways		
REACTOME Up-regulated genes	269	263
REACTOME Down-regulated genes	22	28
REACTOME FDR<0.25 Up-regulated pathways	4	16
REACTOME FDR<0.25 Down-regulated pathways		

Supplementary Table 4: Summary of microarray analysis comparing Fc-GLP-1-treated mice under chow or MCD diet before (T0) and 2 weeks (T14) after partial hepatectomy.

	C+Fc-GLP-1 T0 vs C+Fc-GLP-1 T14	M+Fc-GLP-1 T0 vs M+Fc-GLP-1 T14
KEGG Up-regulated genes	43	1
KEGG Down-regulated genes	91	133
KEGG FDR<0.25 Up-regulated pathways		
KEGG FDR<0.25 Down-regulated pathways	7	69
REACTOME Up-regulated genes	126	2
REACTOME Down-regulated genes	165	289
REACTOME FDR<0.25 Up-regulated pathways	6	
REACTOME FDR<0.25 Down-regulated pathways	36	3

Supplementary Table 5: Specific pathways related to the different gene sets commonly up-regulated in both C+Fc-GLP-1 and M+Fc-GLP-1 groups 2 weeks after partial hepatectomy versus values of the respective liver samples removed in the hepatectomy.

Gene set	Pathways
Cell Cycle	K. Base excision repair<br>K. Cell cycle<br>K. Cytosolic DNA sensing pathway<br>K. DNA replication<br>K. RNA degradation<br>K. RNA polymerase<br>R. Autodegradation of CDH1 by CDH1 APC C<br>R. Activation of ATR in response to replication stress<br>R. Amino acid transport across the plasma membrane<br>R. APC C CDC20 mediated degradation of mitotic proteins<br>R. APC C CDH1-mediated degradation of CDC20 and other APC C CDH1 targeted proteins in late mitosis-early G1<br>R. Assembly of the pre-replicative complex<br>R. CDK-mediated phosphorylation and removal of CDC6<br>R. Cell cycle<br>R. Cell cycle checkpoints<br>R. Cell cycle mitotic<br>R. Cyclin E-associated events during G1 S transition<br>R. DNA repair<br>R. DNA replication<br>R. DNA strand elongation<br>R. G alpha I signalling events<br>R. G alpha Q signalling events<br>R. G1 phase<br>R. G1 S transition<br>R. G2 M checkpoints<br>R. Loss of NLP from mitotic centrosomes<br>R. M G1 transition<br>R. Meiosis<br>R. Meiotic recombination<br>R. Metabolism of nucleotides<br>R. Mitotic G1 G1 S phases<br>R. Mitotic G2 G2 M phases<br>R. Mitotic M M G1 phases<br>R. ORC1 removal from chromatin<br>R. P53 independent G1 S DNA damage checkpoint<br>R. Phase II conjugation

	R. Recruitment of mitotic centrosome proteins and complexes<br>R. Regulation of mitotic cell cycle<br>R. S phase<br>R. Synthesis of DNA
Inflammation	K. B cell receptor signaling pathway<br>K. Chemokine signaling pathway<br>K. Cytokine cytokine receptor interaction<br>K. FC gamma R-mediated phagocytosis<br>K. Hematopoietic cell lineage<br>K. Leukocyte transendothelial migration<br>K. Natural Killer cells-mediated cytotoxicity<br>K. Neurotrophin signaling pathway<br>K. Signaling by ILS<br>K. T cell receptor signaling pathway<br>K. Toll-like receptor signaling pathway<br>R. Activated TLR4 signaling<br>R. Activation of NF KAPPAB in B cells<br>R. Adaptive immune system<br>R. Antigen processing cross presentation<br>R. Chemokine receptors bind chemokines<br>R. Co-stimulation by the CD28 family<br>R. Cytokine signaling in immune system<br>R. Downstream signaling events of B cell receptor BCR<br>R. Gamma R mediated phagocytosis<br>R. IL 2 signaling<br>R. IL 3, 5 and GM CSF signaling<br>R. IL1 signaling<br>R. Immune system<br>R. Immunoregulatory interactions between a lymphoid and a non-lymphoid cells<br>R. Innate immune system<br>R. Interferon alpha beta signaling<br>R. Interferon gamma signaling<br>R. Interferon signaling<br>R. NOD1, 2 signaling pathway<br>R. Semaphorin interactions<br>R. Signaling by ILs<br>R. Signaling by the B cell receptor BCR<br>R. TCR signaling
HSC and EM remodelling	K. Adherens junctions<br>K. Cell adhesion molecules cams<br>K. Notch signaling pathway<br>K. Regulation of actin cytoskeleton<br>K. Retinol metabolism<br>K. TGF beta signaling pathway<br>K. Tight junction

	K. Wnt signaling pathway<br>R. Actin cytoskeleton<br>R. Cell-cell communication<br>R. Tight junction organization<br>R. Cell junction organization<br>R. Collagen formation<br>R. Degradation of the extracellular matrix<br>R. Extracellular matrix organization<br>R. Integrin cell surface interactions<br>R. Pre-Notch expression and processing<br>R. Signaling by Notch<br>R. Signaling by TGF beta receptor complex<br>R. Signaling by Wnt<br>R. Tight junction interactions
Metabolism	K. Amino sugar and nucleotide sugar metabolism<br>K. Arachidonic acid metabolism<br>K. Fatty acid metabolism<br>K. Galactose metabolism<br>K. Glutathione metabolism<br>K. Glycerolipid metabolism<br>K. Metabolism of xenobiotics by cytochrome P450<br>K. Purine metabolism<br>K. Pyrimidine metabolism<br>K. Starch and sucrose metabolism<br>K. Tyrosine metabolism<br>R. Biological oxidations<br>R. Diabetes pathways<br>R. Glutathione conjugation<br>R. Glycosphingolipid metabolism<br>R. Lipid digestion mobilization and transport<br>R. Metabolism of amino acids and derivatives<br>R. Metabolism of carbohydrates<br>R. Phospholipid metabolism<br>R. PI metabolism<br>R. Purine metabolism
Apoptosis	K. ABC transporters<br>K. Apoptosis<br>K. Mismatch repair<br>K. P53 signaling pathway<br>R. Apoptosis<br>R. Apoptotic cleavage of cellular proteins<br>R. Apoptotic execution phase<br>R. Cell death signaling via NRAGE NRIF and NADE<br>R. Chromosome maintenance<br>R. Destabilization of mRNA by AUF1 HNRNP D0<br>R. ER phagosome pathway

	R. Intrinsic pathway for apoptosis<br>R. P53 dependent G1 DNA damage response<br>R. Regulation of apoptosis
Hemostasis	K. Complement and coagulation cascades<br>K. VEGF signaling pathway<br>R. Factors involved in megakaryocyte development and platelet production<br>R. Hemostasis<br>R. Heparan sulfate heparin HS GAG metabolism
GPCR	R. GPVI mediated activation cascade<br>R. Signaling by GPCR<br>R. GPCR ligand binding<br>R. Peptide ligand binding receptors<br>R. Class A1 rhodopsin-like receptors<br>R. GPCR downstream signaling<br>R. Signaling by RHO GTPases
ER stress and Proteasome	K. JAK STAT signaling pathway<br>K. Ubiquitin mediated proteolysis<br>K. Proteasome<br>R. Activation of chaperone genes by XBP1S<br>R. Autodegradation of the E3 ubiquitin ligase COP1<br>R. Protein folding<br>R. Antigen processing ubiquitination proteasome degradation<br>R. SCFSKP2-mediated degradation of P27 P21

R; Reactome database. K; KEGG database.

LEGENDS TO SUPPLEMENTARY FIGURES

Supplementary Figure 1: **A)** Experimental design for the study of treatment with Fc-GLP-1 in a non-obese model of NASH and hepatic regeneration in mice. **B)** Experimental design for the study of treatment with Fc-GLP-1 in an obese model of NASH and hepatic regeneration in mice.

Supplementary Figure 2: Plasma levels of Fc-GLP-1 sampled 48 h after an injection during the liver regeneration phase (n=6 mice/group).

Supplementary Figure 3: Insulin plasma levels in different experimental groups two weeks after PH. (n=6 mice/group). ****$p<0.001$ vs C group according to one way ANOVA with post-hoc Bonferroni test.*

Supplementary Figure 4: Gene sets significantly down-regulated (FDR <0.25) in M+Fc-GLP-1 treated mice versus MCD diet-fed animals in the microarray analysis (n=4 mice/group). The analysis was performed 2 weeks after PH.

Supplementary Figure 5: Changes of intrahepatic glycogen levels in the experimental groups. **$p<0.05$, ***$p<0.001$ vs C group according to one way ANOVA with post-hoc Bonferroni test (n=6-8 mice/group)*

Supplementary Figure 6: **A)** Experimental study design for the effect of treatment with Liraglutide in a non-obese model of NASH and hepatic regeneration in mice. **B)** Survival ratio after PH in different animal groups. **C)** Representative images of H&E staining from mice fed with standard chow diet (C) or MCD diet untreated or treated with Liraglutide (C+Lira, M+Lira). **D)** Effects of liraglutide on ALT plasma levels after PH. **E)** Regeneration ratio determined at 2 weeks post-PH. ***$p<0.01$, ***$p<0.001$ vs C group according to one way ANOVA with post-hoc Bonferroni test (n=6 mice/group).*

Supplementary Figure 7: TEM images of liver sections from chow and MCD diet-fed mice after PH showing lipid droplets (LD), hepatic stellate cells (SC), Kupffer cells (KC) in the sinusoidal space (SS) and recruited immune cells (RIC).

Supplementary Figure 8: **A)** Gene sets significantly up-regulated (FDR<0.25) in livers of C+Fc-GLP-1-treated animals after PH versus values obtained from the same group of mice in liver samples removed in the hepatectomy surgery (n=4 mice/group). **B)** Gene sets groups significantly up-regulated (FDR<0.25) in M+Fc-GLP-1 mice under conditions described above *(n=4 mice/group).*

Supplementary Figure 9: Mice were fed a HFD for 10 weeks and then divided into 2 groups as follows: mice that continued receiving a HFD and injected vehicle (HFD) and mice fed a HFD treated with Fc-GLP-1 (H+Fc-GLP-1) for a further 3 weeks. Then, PH was performed and mice were sacrificed after 2 weeks. Evolution of body weight along the experimental protocol *(n=5-6 mice/group).*

Supplementary Figure 10: Heat maps corresponding to up-regulated gene families related with remodeling of the ECM in M+Fc-GLP-1 animals 2 weeks after PH compared with values obtained from the same group of mice in liver samples removed in the hepatectomy.

Supplementary Figure 11: Heat maps corresponding to up-regulated gene families related with adaptive immune responses in M+Fc-GLP-1 animals 2 weeks after PH compared with values obtained from the same group of mice in liver samples removed in the hepatectomy.

A)

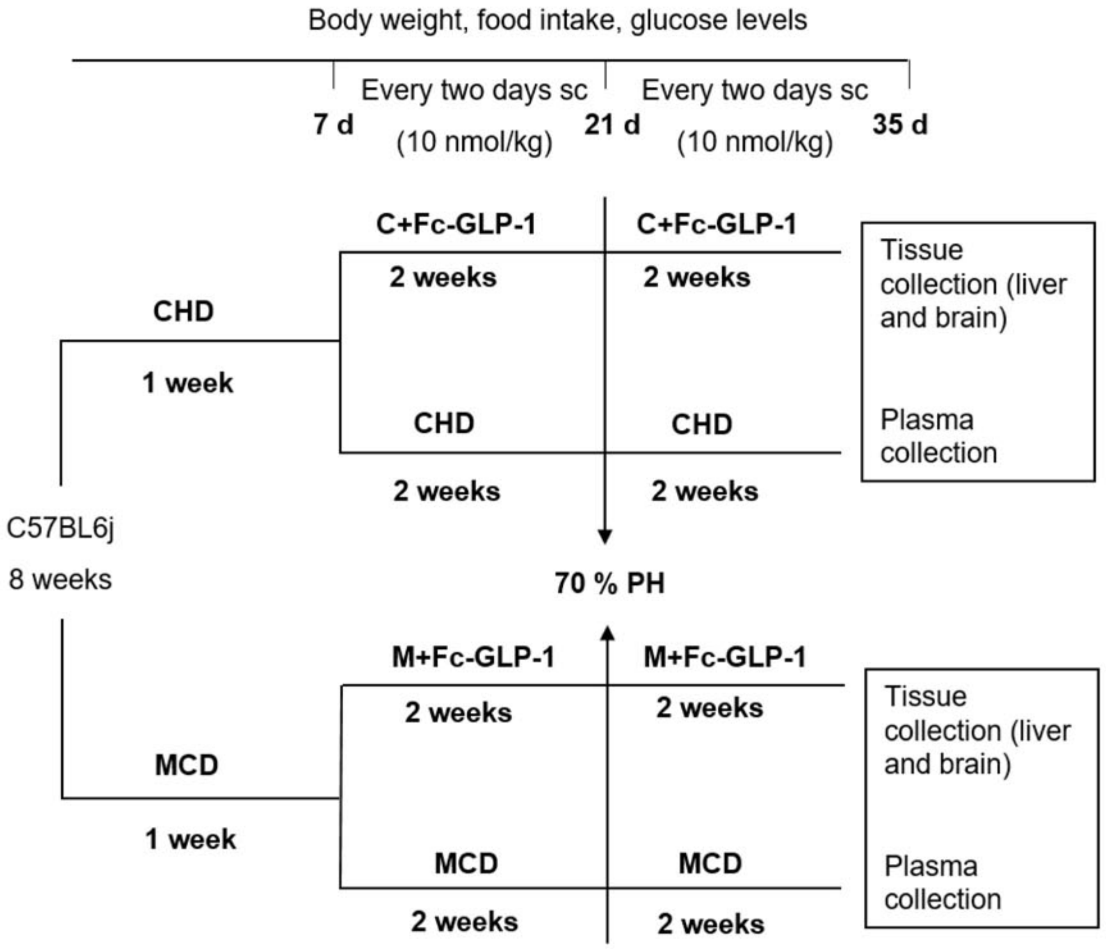

B)

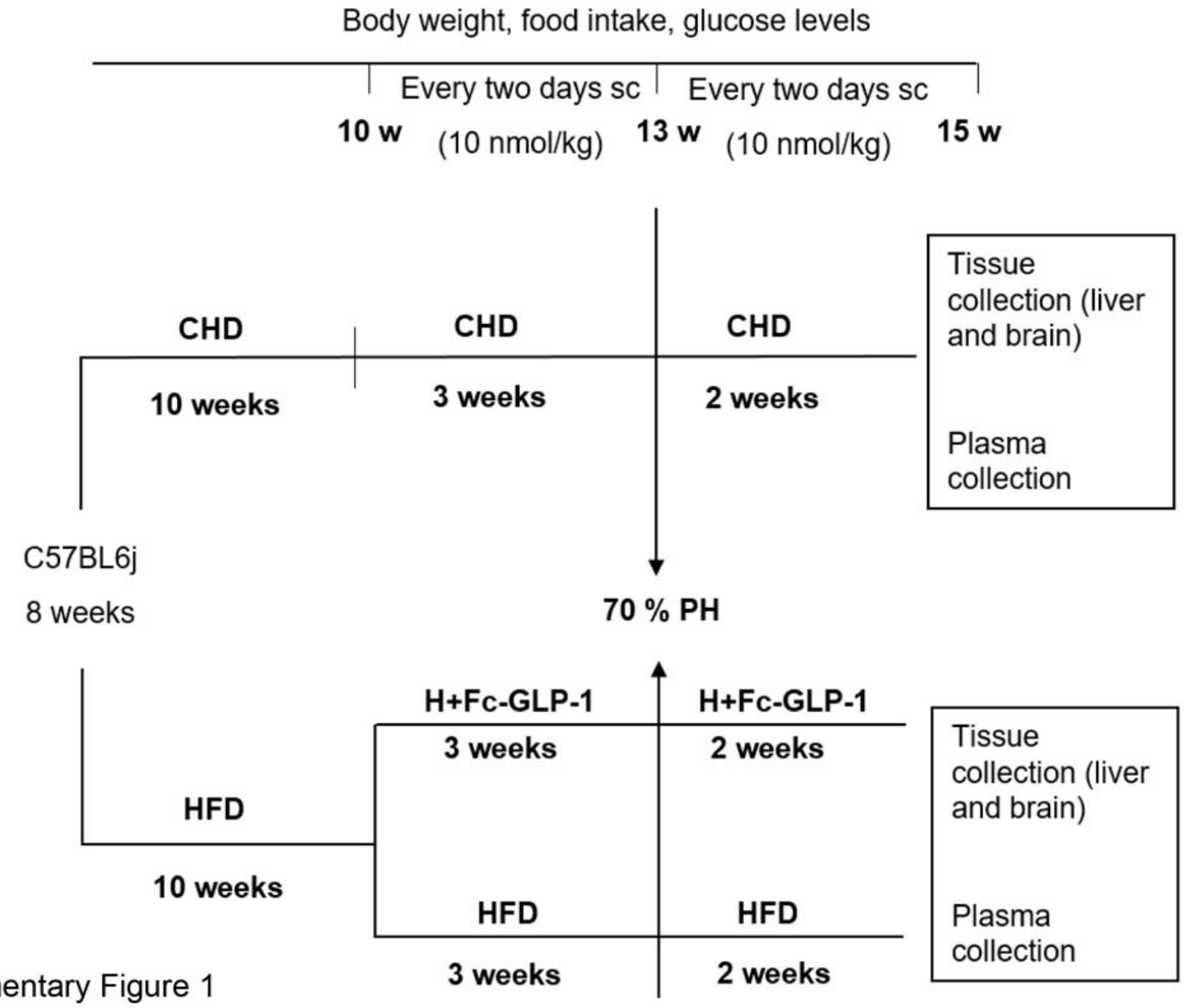

Supplementary Figure 1

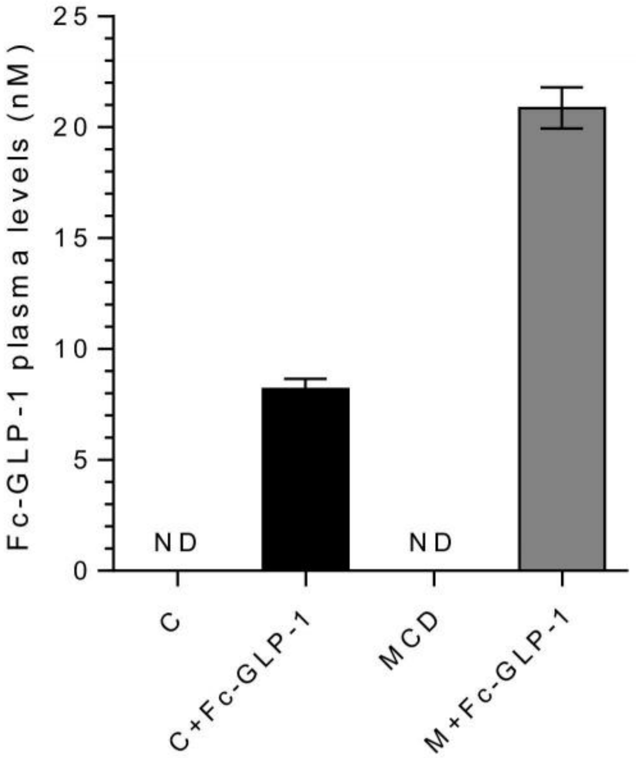

ND= non-determined

Supplementary Figure 2

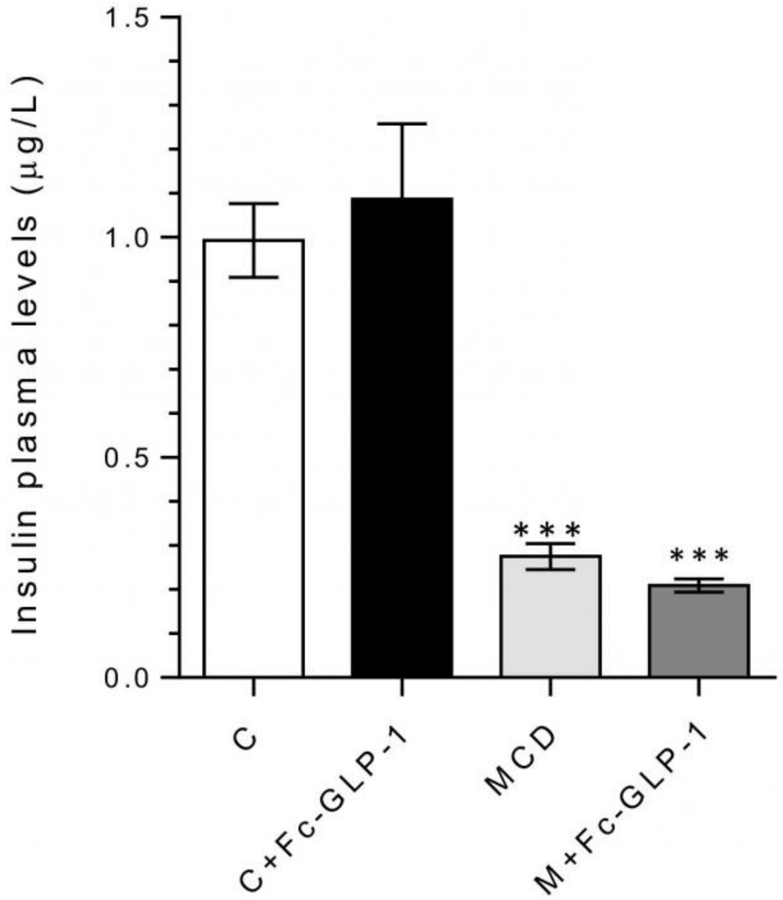

Supplementary Figure 3

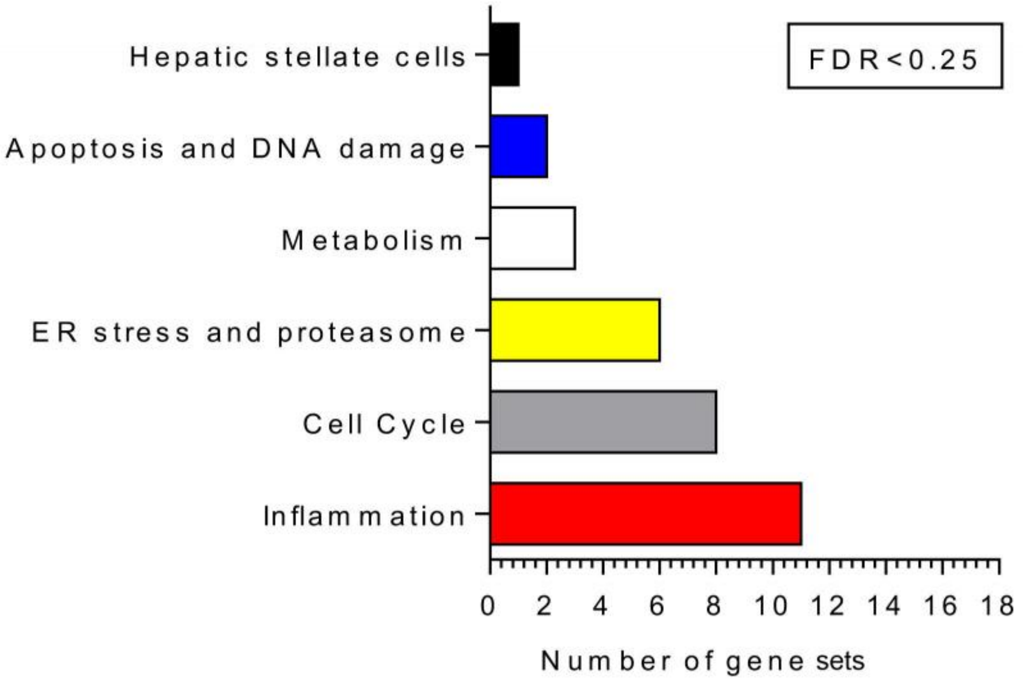

Supplementary Figure 4

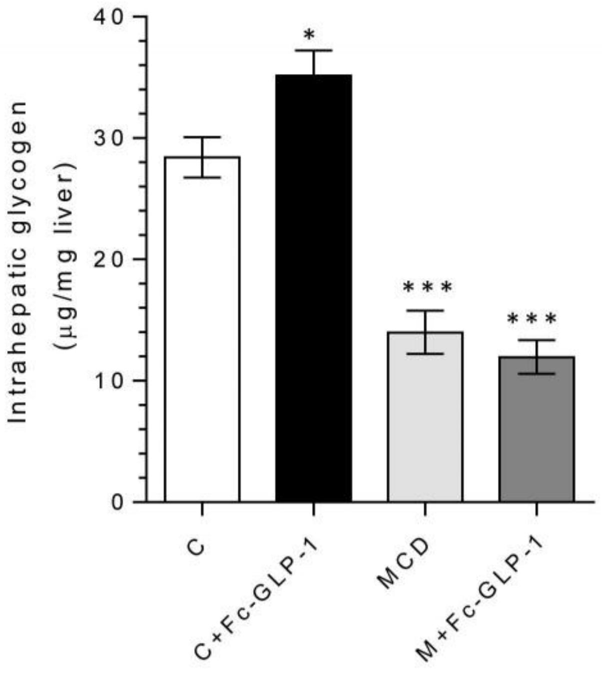

Supplementary Figure 5

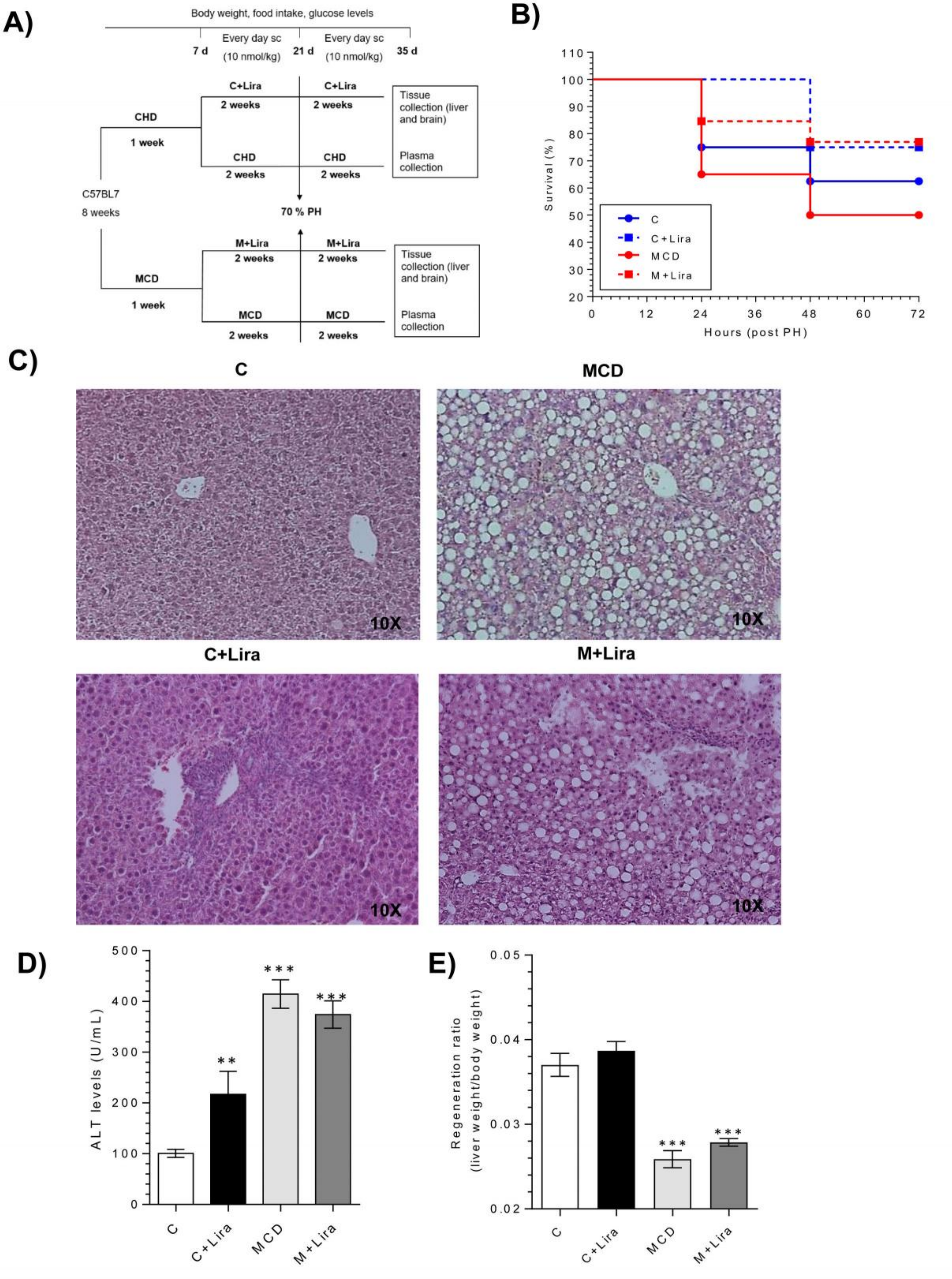

Supplementary Figure 6

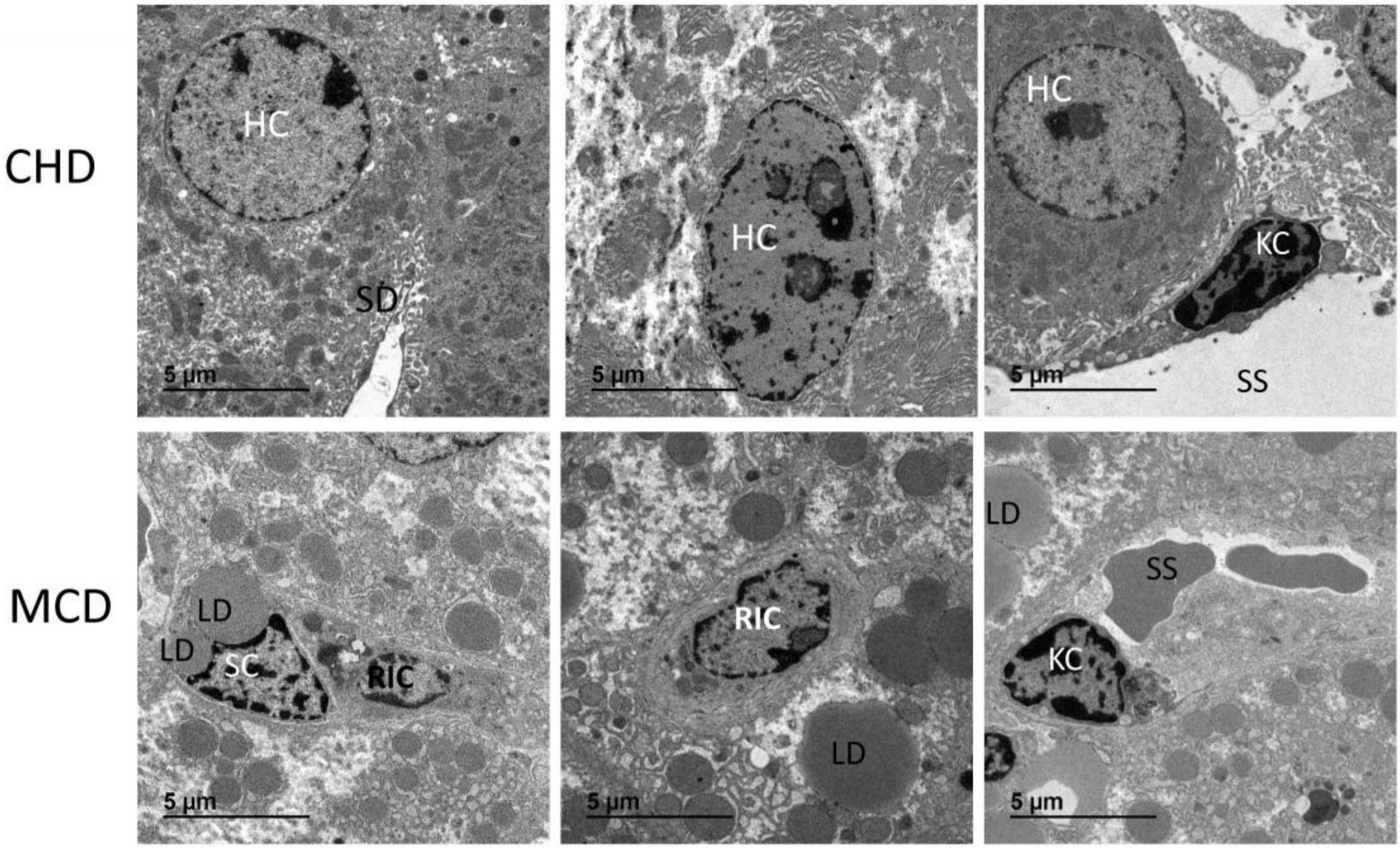

Supplementary Figure 7

A)

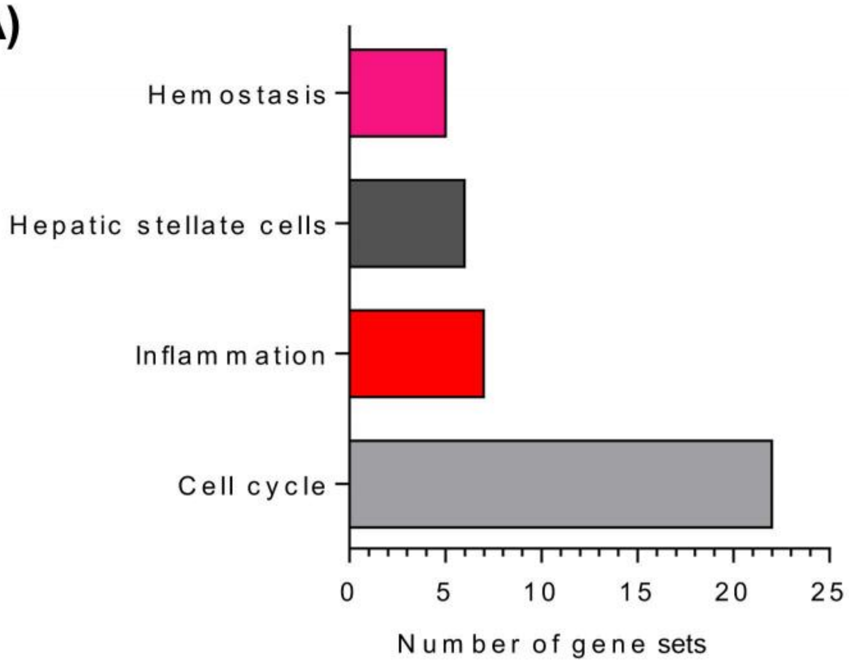

B)

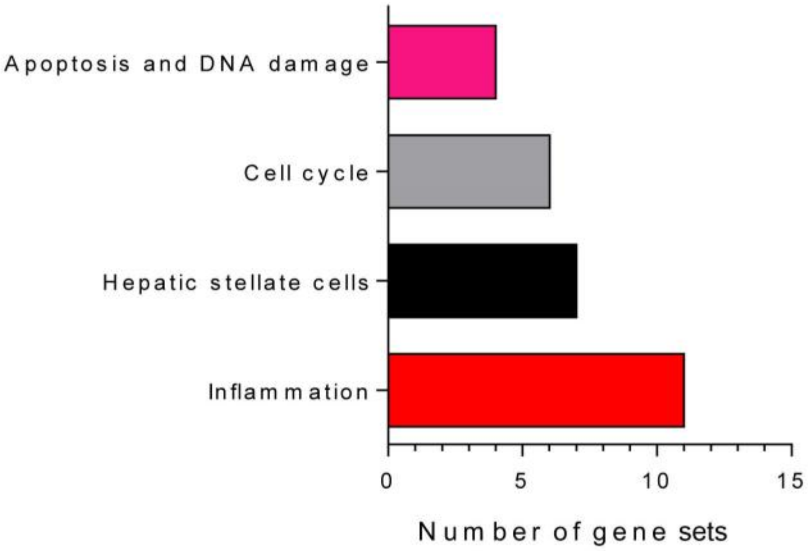

Supplementary Figure 8

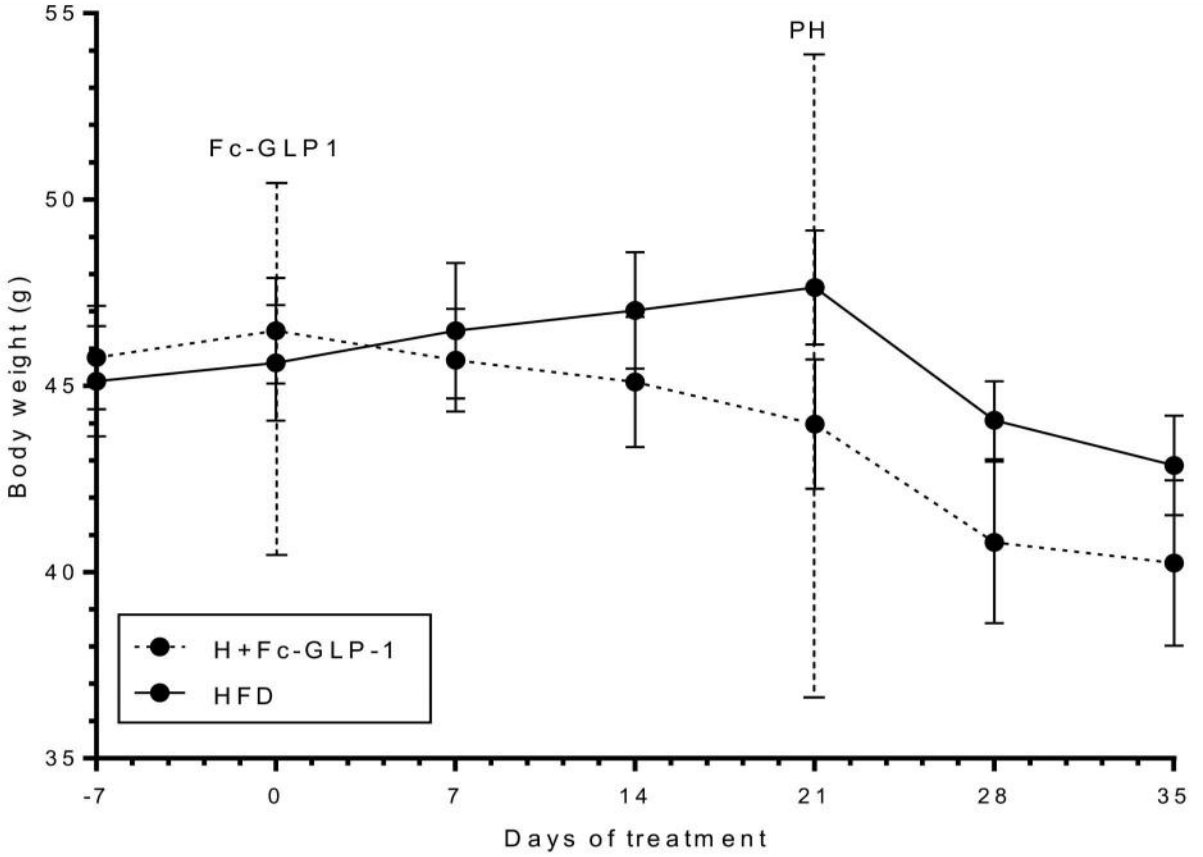

Supplementary Figure 9

KEGG ECM receptor interaction

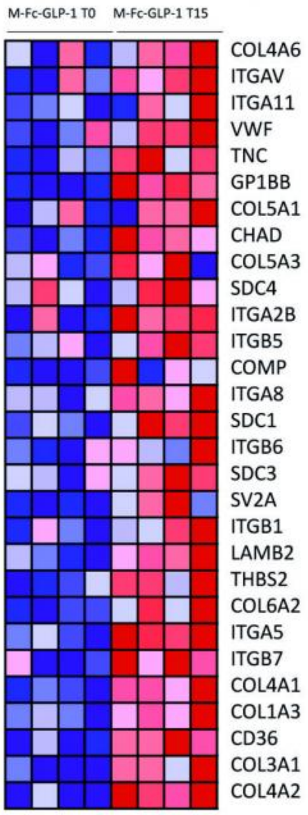

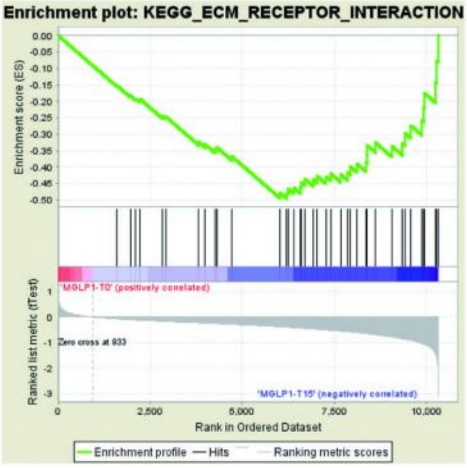

KEGG regulation of actin cytoskeleton

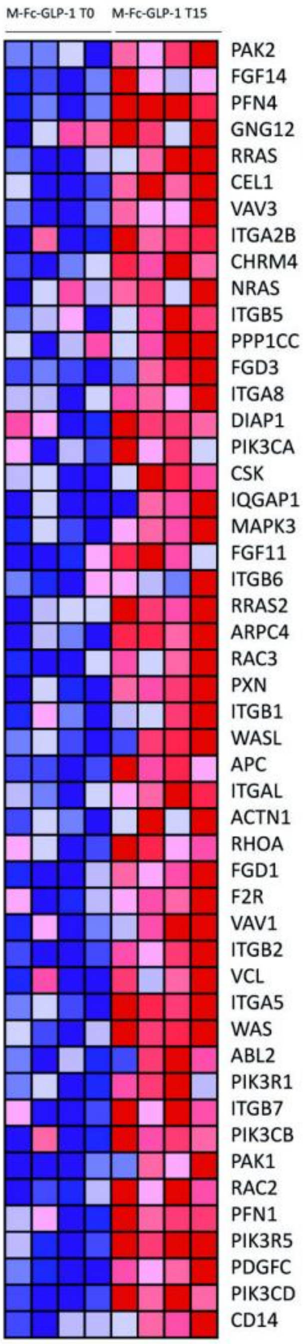

KEGG TGFβ signalling pathway

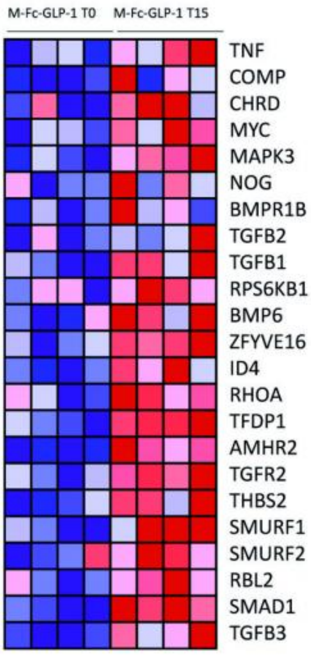

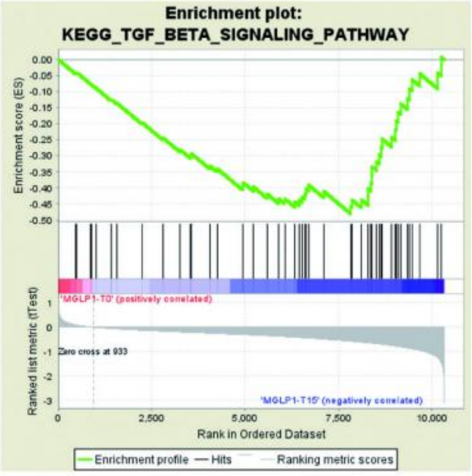

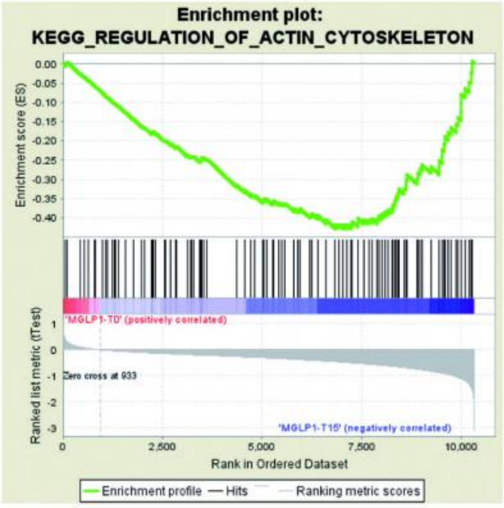

Supplementary Figure 10

KEGG Natural Killer cell mediated cytotoxicity

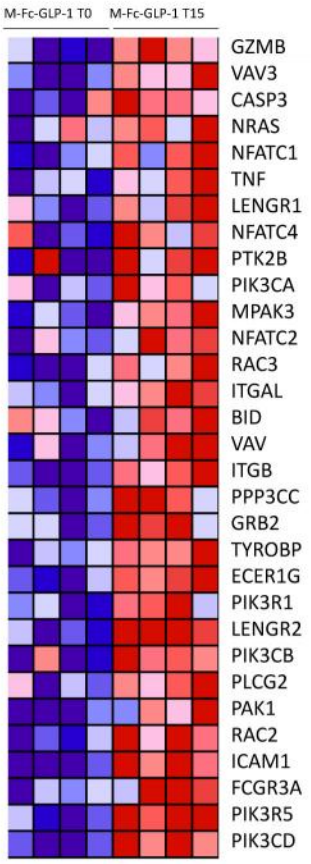

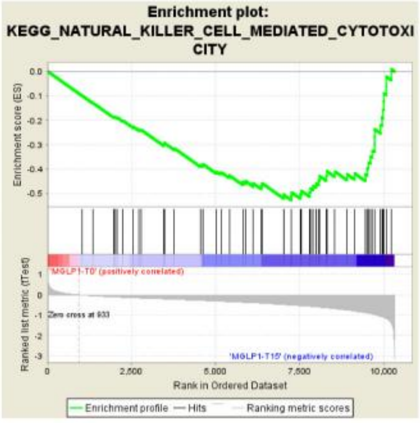

KEGG Cytokine cytokine receptor interaction

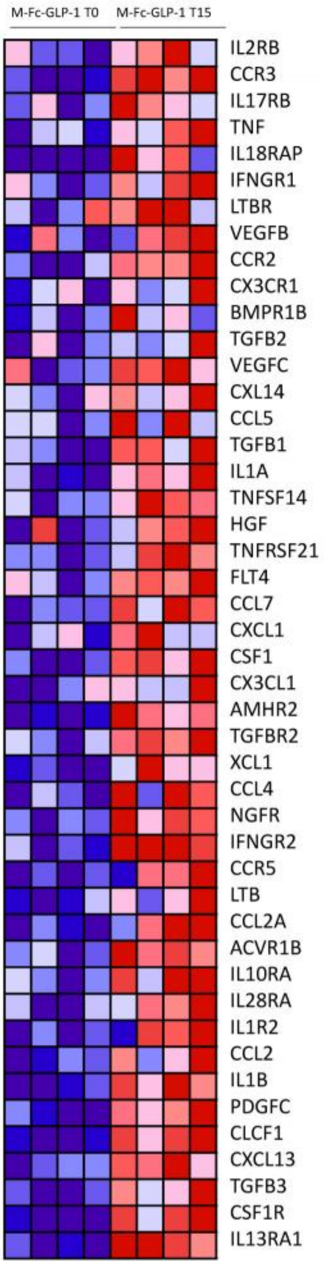

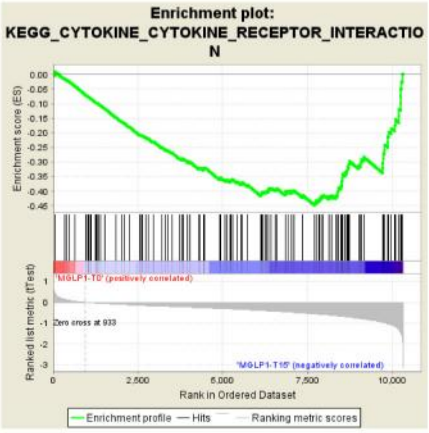

KEGG Leukocyte transendothelial migration

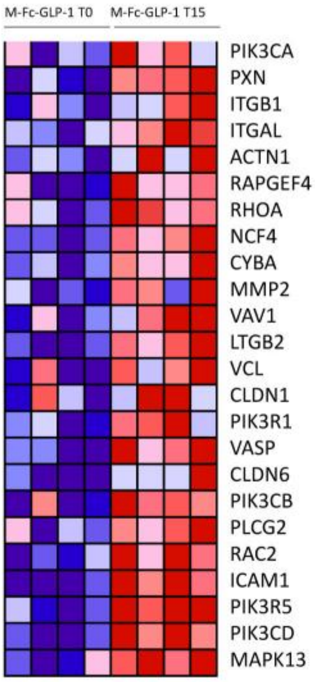

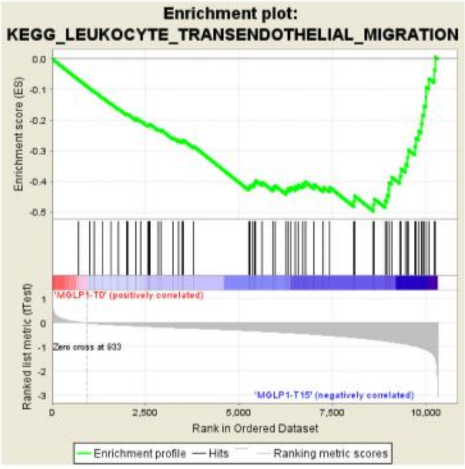

Supplementary FIgure 11
